# The effectiveness of physical activity interventions using activity trackers during or after inpatient care: a systematic review and meta-analysis of randomized controlled trials

**DOI:** 10.1186/s12966-022-01261-9

**Published:** 2022-05-23

**Authors:** Marijke E. de Leeuwerk, Petra Bor, Hidde P. van der Ploeg, Vincent de Groot, Marike van der Schaaf, Marike van der Leeden, on behalf of the OPRAH consortium, Edwin Geleijn, Vincent van Vliet, Sven J. G. Geelen, Rosalie J. Huijsmans, Hinke M. Kruizenga, Peter J. M. Weijs, Suzanne ten Dam, Marc G. Besselink, Chris Dickhoff, Jurriaan B. Tuynman, Mark I. van Berge Henegouwen, Anne M. Eskes, Mirjam A. G. M. Pijnappels, Johannes C. F. Ket, Martijn W. Heijmans

**Affiliations:** 1 Amsterdam UMC location Vrije Universiteit Amsterdam, Rehabilitation Medicine, de Boelelaan, 1117 Amsterdam, the Netherlands; 2 Amsterdam Movement Sciences, Ageing & Vitality, Amsterdam, The Netherlands; 3 University Medical Centre Utrecht, Utrecht University, Department of Rehabilitation, Physical Therapy Science & Sports, Heidelberglaan, 100 Utrecht, the Netherlands; 4 Amsterdam UMC location Vrije universiteit Amsterdam, Public and Occupational Health, de Boelelaan, 1117 Amsterdam, the Netherlands; 5 Amsterdam Public Health, Health Behaviors & Chronic Diseases, Amsterdam, The Netherlands; 6 Amsterdam Movement Sciences, Rehabilitation & Development, Amsterdam, The Netherlands; 7 Amsterdam UMC location University of Amsterdam, Rehabilitation Medicine, Meibergdreef 9, Amsterdam, the Netherlands; 8 Faculty of Health, Center of Expertise Urban Vitality, Amsterdam University of Applied Sciences, Amsterdam, the Netherlands

**Keywords:** Physical activity, Physical functioning, Activity tracker, Hospitalization, Rehabilitation

## Abstract

**Background:**

Promoting physical activity (PA) in patients during and/or after an inpatient stay appears important but challenging. Interventions using activity trackers seem promising to increase PA and enhance recovery of physical functioning.

**Objective:**

To review the effectiveness of physical activity interventions using activity trackers on improving PA and physical functioning, compared to usual care in patients during and/or after inpatient care. In addition, it was determined whether the following intervention characteristics increase the effectiveness of these interventions: the number of behaviour change techniques (BCTs) used, the use of a theoretical model or the addition of coaching by a health professional.

**Design:**

Systematic review and meta-analysis.

**Data Sources:**

PubMed, EMBASE, Cinahl, SportDiscus and Web of Science databases were searched in March 2020 and updated in March 2021.

**Eligibility criteria for selecting studies:**

Randomized controlled trials (RCTs) including interventions using activity trackers and feedback on PA in adult patients during, or less than 3 months after, hospitalization or inpatient rehabilitation.

**Methods:**

Following database search and title and abstract screening, articles were screened on full text for eligibility and then assessed for risk of bias by using the Physiotherapy Evidence Database (PEDro) scale. Meta-analyses, including subgroup analysis on intervention characteristics, were conducted for the outcomes PA and physical functioning.

**Results:**

Overall, 21 RCTs totalling 2355 patients were included. The trials covered a variety of clinical areas. There was considerable heterogeneity between studies. For the 13 studies that measured PA as an outcome variable(*N* = 1435), a significant small positive effect in favour of the intervention was found (standardized mean difference (SMD) = 0.34; 95%CI 0.12–0.56). For the 13 studies that measured physical functioning as an outcome variable (*N* = 1415) no significant effect was found (SMD = 0.09; 95%CI -0.02 - 0.19). Effectiveness on PA seems to improve by providing the intervention both during and after the inpatient period and by using a theoretical model, multiple BCTs and coaching by a health professional.

**Conclusion:**

Interventions using activity trackers during and/or after inpatient care can be effective in increasing the level of PA. However, these improvements did not necessarily translate into improvements in physical functioning. Several intervention characteristics were found to increase the effectiveness of PA interventions.

**Trial registration:**

Registered in PROSPERO (CRD42020175977) on March 23th, 2020.

**Supplementary Information:**

The online version contains supplementary material available at 10.1186/s12966-022-01261-9.

## Introduction

Admission to a hospital or rehabilitation centre often leads to a decline in physical functioning [1–4]. This may be caused by the initial disease or medical treatment, but also by a reduction in physical activity (PA). It has been shown that increasing PA during or after inpatient care is effective in improving recovery in physical functioning [2, 5–8]. However, stimulating PA in patients during and after an inpatient stay appears to be challenging because healthcare professionals may have insufficient time and patients may experience physical discomfort or lack of motivation [9–12]. Therefore, extra support to increase PA levels is desired [13].

Activity trackers are wearable devices to monitor PA and are commonly used in interventions to stimulate PA [14–18]. In various patient populations, for example in patients with COPD or with rheumatic and musculoskeletal diseases, the use of activity trackers was found effective in increasing PA [14–18]. The evidence of effectiveness of interventions with activity trackers on physical functioning has been studied less and is conflicting [16, 17].

The use of interventions with activity trackers during or after inpatient care is expected to be effective, because an inpatient period, for example after oncological surgery or after a neurological event, can be considered as a “teachable moment”: a time frame following a health event which a patients is most conducive to behavioural change [19, 20]. However, the effectiveness of PA interventions with activity trackers during or after admission to a hospital or rehabilitation centre has not been summarized systematically to date.

There is a wide variation in interventions with activity trackers. It is therefore important to identify which intervention characteristics have the highest effect on increasing patients’ PA. To systematically describe, develop and test active elements of behavioural health interventions a taxonomy of behaviour change techniques (BCTs) has been developed [21]. BCTs are “observable, replicable and irreducible components of an intervention designed to alter or redirect causal processes that regulate behaviour” [21]. Interventions with activity trackers often contain several BCTs [22]. However, there is insufficient evidence about the potential for the use of BCTs to improve the effectiveness of an intervention in patients during or after inpatien care.

Besides BCTs, there is evidence for the use of a theoretical model, e.g. the Trans theoretical Model (TTM), the Social Cognitive Theory (SCT) or the self-efficacy theory [23–26]. Theory-based interventions are expected to be more effective because they tend to be better substantiated and more carefully described and carried out. In addition, the engagement of coaching from a health professional during the intervention may also influence the impact on the targeted behaviour (PA) [27]. It is expected that a health professional having insight into the level of PA will be more motivating to the patient and PA goals can be better adjusted by the health professional during the intervention.

The primary aim of this study was to review the effectiveness of physical activity interventions using activity trackers on PA and physical functioning, compared to usual care in patients during or after inpatient care. The secondary aim was to determine whether the following intervention characteristics increase the effectiveness of these interventions: the number of BCTs used, the use of a theoretical model or the addition of coaching by a health professional.

## Methods

### Protocol and registration

The review protocol was registered in the International Prospective Register of Systematic Reviews (PROSPERO) at https://www.crd.york.ac.uk/prospero/ (registration number CRD42020175977, submitted on March 23th, 2020). This review applies a systematic approach according to the PRISMA (Preferred Reporting Items for Systematic Reviews and Meta-Analyses) updated guideline [28].

### Search strategy

A systematic literature search was conducted in March 2020 and updated on 3 March 2021, using the databases PubMed, EMbase.com, Ebsco/CINAHL, Ebsco/SportDiscus and Clarivate Analytics/Web of Science Core Collection (by MEL and JCFK). The search strategy included the following search terms and their synonyms: (1) inpatient period, (2) activity trackers and (3) adult patients. The full search string is presented in Electronic Supplementary Material Table S1. The reference lists of the included studies were checked to detect additional articles.

### Study selection

The software program ‘Rayyan’ was used for the study selection. The studies were independently screened by two reviewers (ML and PB), first on title and abstract and second on full text, to assess eligibility for inclusion. The reviewers were blinded to each other’s decisions. If necessary, final judgement about the eligibility was made by a third reviewer (MvdL).

### Eligibility criteria

#### Type of studies

Randomized controlled trials about interventions with the use of activity trackers and feedback on PA level were included. No restrictions concerning the language or year of publication were used.

#### Type of participants

The target population for this review were adults during or less than 3 months after hospitalization or inpatient rehabilitation. No restrictions were made for the medical reason of the inpatient period.

#### Type of intervention

All studies with an intervention that included (1) an objective measurement of PA with the use of an activity tracker (e.g. accelerometer or pedometer) and (2) feedback on PA level for the participant (e.g. visual feedback from the activity tracker or feedback from a therapist), alone, or in combination with other interventions, were included. Studies that only used activity trackers to measure activity of the upper body were excluded from this review.

#### Type of control group

Usual care or an intervention with activity trackers without any form of feedback on PA level.

#### Type of outcomes

The main outcomes of this review were PA and physical functioning. For this study, we used the definition of physical activity defined by the World Health Organization (WHO), i.e. any bodily movement produced by skeletal muscles that requires energy expenditure [29]. Up until now there is no consensus on the definition of physical functioning. For this study, physical functioning was defined as the ability to perform both basic and instrumental activities of daily living, this definition is more often used in other studies [30]. Studies were eligible if they had included an objectively measured outcome of PA (i.e. steps per day or active minutes per day) or if they had measured physical functioning by means of performance-based measures or by patient-reported measures (PROM) of function.

### Data extraction

The following study characteristics were extracted from the included RCTs: author, year of publication, study population, group characteristics, setting, description of the intervention, intervention characteristics, description of the control group and outcome measures of the primary outcomes for this review. The following intervention characteristics were extracted: duration, coaching by a health professional during the intervention (yes/no), theory mentioned (e.g. social cognitive theory)(yes/no) and type of activity tracker. If an article reported multiple comparisons, we only extracted data from the groups of interest. For the outcome PA, we extracted steps per day if available. We had chosen for steps/day because this is the most common used outcome for PA and is currently the most convenient to interpret. When this data was not available, we extracted another outcome measured with the accelerometer (e.g. active minutes per day). For the outcome physical functioning, we had chosen to extract the most task-specific test (e.g. Short Physical Performance Battery rather than a muscle strength test), because task-specific tests are more indicative of patients ADL-functioning. The data was extracted by one reviewer and verified by a second reviewer. Disagreements were resolved by discussion.

### Coding of behaviour change techniques

The BCT taxonomy (v1) of 93 hierarchically cluster techniques from Michie et al. was used to identify and code the BCTs reported in the intervention [21]. The most comprehensive description of the intervention was used (e.g. study protocol). Coding was carried out by one reviewer (ML) and a second independent reviewer (PB) double coded a random 20% of all descriptions to check for reliability. Disagreements were resolved via discussion. Cohen’s kappa was used to measure the agreement between the reviewers. Both reviewers completed the BCT taxonomy v1 Online Training. The BCTs in the intervention and control group were identified separately and only the BCTs exclusively used in the intervention group were extracted. In addition, the total number of BCTs used in the intervention were recorded.

### Evaluation of the methodological quality

The Physiotherapy Evidence Database (PEDro) scale was used to assess the methodological quality of the individual studies. The PEDro scale is a valid and reliable tool for assessing methodological quality of clinical trials and randomized controlled trials [31, 32]. The PEDro scale consists of 11 items; eight items (item 2–9) are used to asses internal validity and two (item 10–11) items are used to assess interpretability of the results. The first item, which assesses the external validity, is excluded in calculating the total score (following the methods of the PEDro score) [33]. Therefore, the score ranges from 0 to 10 points. A higher score indicates a lower risk of bias. Trials with a score of ≥6 were considered as ‘low risk’ of bias. Trials were considered as ‘high risk’ of bias if they had a score < 6 [32]. Quality assessment was independently conducted by two reviewers. Disagreement between the reviewers was discusses with a third reviewer (MvdL). Cohen’s kappa was used to measure the agreement between the reviewers.

### Data analysis

Outcomes of the studies were collected at baseline, during the intervention, post-intervention (within 1 month after the end of the intervention period) and long term follow up if available. Outcomes not included in the meta-analyses were presented descriptively.

#### Meta-analysis

A meta-analysis was conducted for the post-intervention outcomes of PA and physical functioning. The studies varied in the use of statistics and reporting of the effect sizes. The mean difference and standard deviation (SD) between baseline and post-intervention were extracted. If not reported in the study results, the mean difference and SD were calculated. In case data was missing to calculate the mean difference, authors were contacted. If only median and interquartile ranges (IQR) were reported, the sample mean and standard deviation were estimated following the method of Wan et al. [34].

The software program Review Manager (version 5.3.5) was used to conduct the meta-analysis. Included studies were assessed on statistical and clinical heterogeneity by inspection of the forest plots and the I^2^ statistics. If no considerable between-group statistical or clinical heterogeneity was detected, the fixed effects model was used; otherwise, a random effects model was used. Meta-analysis was performed to calculate the pooled treatment effect size with a 95% confidence interval for both outcomes. Results were visually presented using forest plots. An effect size of 0.2 was considered as small, 0.5 as moderate and 0.8 or higher as large [35]. A funnel plot and Egger’s regression test was used to assess the presence of publication bias. If Egger’s regression test shows a significance level ≤ 0.05, there is a high probability of publication bias. Leave-one-out sensitivity analysis was conducted in order to confirm that the results were not driven by any single study.

#### Subgroup-analyses

For this review a broad population has been included, therefore the different study populations were expected to be heterogeneous. To explore the contribution of different study characteristics on the overall outcome, pre-specified subgroup analyses were conducted for the following possible moderators: (1) setting (hospitalization vs rehabilitation), (2) period of intervention (during and/or after the inpatient period), (3) duration of the intervention (≤3 months or > 3 months) and the age group of the participants (mean age ≤ 60 years or mean age > 60 years). In addition, subgroup analyses were performed on methodological quality (low risk of bias vs. high risk of bias) to explore if the methodological quality has affected the overall effect size. Cochrane’s Q test was performed to test whether there was a significant moderation effect (*p* < 0.05).

Given the small number of included studies and the large variety in combination of coded BCTs, it was not possible to determine the effect of combinations of different BCTs using meta-regression. It was decided not to perform sub-analysis of individual BCTs, because it is suggested that a combination of different BCTs is more important than the effect of a single BCT [36]. Therefore, subgroup analyses were conducted in the following intervention characteristics: (1) number of BCTs used in the intervention, theory-based interventions (yes/no) and (3) coaching by a health professional (yes/no). The cut-off value for the subgroup analysis of the number BCTs was determined by the mean number of BCTs used in the included studies. In addition, it was investigated how the use of BCTs differed between these subgroups.

## Results

### Study selection

**Fig. 1:**
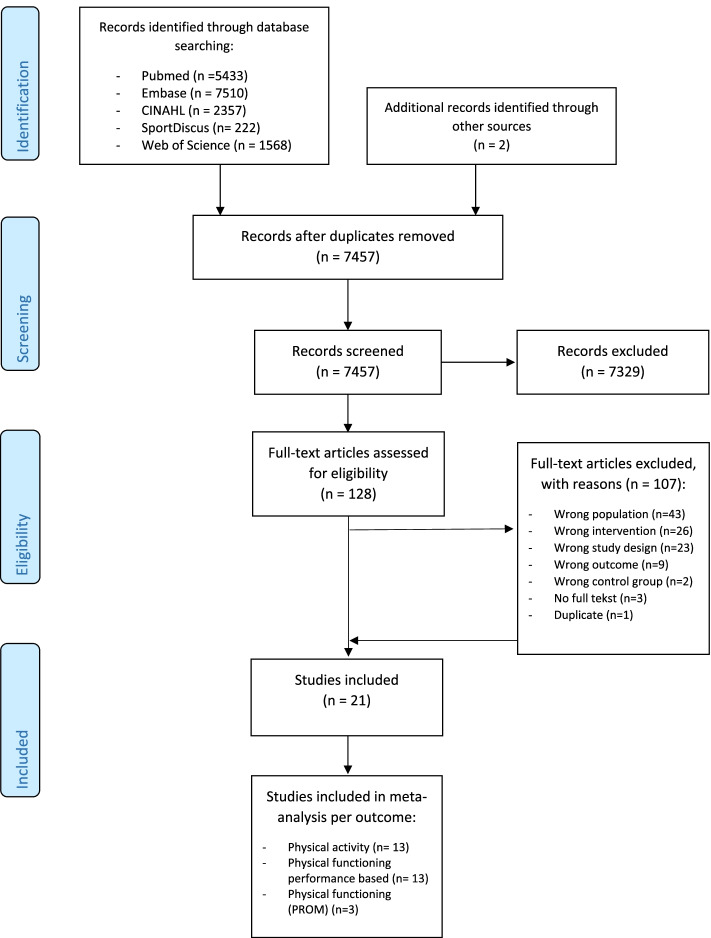
Flow diagram of selected studies (PRISMA)

After removing duplicates from the initial search, a total of 7457 articles were screened on title and abstract. Of the 128 articles screened on full-text, 107 articles were excluded. Reasons for exclusion are shown in the flow diagram (Fig. 1). A total of 21 RCTs were included in this review, totalling 2355 patients.

### Study characteristics

With the exception of the study of Izawa et al. (2005) [37], all trials were published between 2011 and 2020. The number of participants per study ranged from 30 to 344. The following patient populations were present in the included studies: patients with neurological diseases [38–42], patients with cardiovascular diseases [37, 43–45], patients after orthopaedic surgery [46–50], patients after abdominal surgery [51, 52], oncological patients [53], patients with COPD [54], patients after bariatric surgery [55], older patients admitted to post-acute care rehabilitation [56] and patients with low functional independence [57]. Eight trials were performed during the inpatient period, eight after the inpatient period and five trials both during and after the inpatient period. Eleven trials were performed during and/or after hospitalization, ten trials were performed during and/or after inpatient rehabilitation. Other study characteristics are presented in Table 1.

**Table 1:** Characteristics of included RCT’s

Author (year)	Population	Group characteristics, sample size; n, male; n(%), age; mean ±SD	Setting	Intervention					Control	PA outcome measure(s)^a^	PF performance-based outcome measure(s)^a^	PF patient reported outcome measure^a^	Short conclusion
				Descriptive	duration	Coaching by a health professional	Theory used	Type of activity tracker					
Atkins (2019) [57]	Patients with lower initial function independence measure scores and longer anticipated length of stay.	Intervention: n = 39, 20 (51), 74 ± 17 Control: n = 39, 12 (31), 78 ± 18	During inpatient rehabilitation	Usual care + pedometer with feedback on step count	1 month*	No	NA	Yamax Digiwalker SW200 pedometer	Usual care + pedometer without feedback on step count.	^a^**Steps/day (D)** Daily upright time	^a^**Morton mobility index (DEMMI) (P)**	NA	Pedometers without targets do not improve functional mobility
Brandes (2018) [46]	Patients after primary, unilateral joint replacement due to knee or hip osteoarthritis	Intervention: n = 23, 11 (48), 71 ± NA Control: n = 26, 12 (46), 70 ± NA	During inpatient rehabilitation	Usual care + activity tracker with physical activity counselling with tailored approach by adding +5% in daily steps compared to the previous days	3 weeks*	Yes (RL)	NA	Step Activity Monitor 3.0	Usual care	^a^**Steps/day** ^a^**(P, FU)** Active minutes/day Inactive time	NA	^a^**Oxford hip/knee score (P, FU)**	PA counselling during inpatient rehabilitation did not improve PA or functional outcomes
Christiansen (2020) [49]	Patients after a unilateral total knee replacement	Intervention: n=20, 12 (60), 66.5 ± 6.9 Control: n=23, 8 (35), 67.5 ± 7.2	After hospital discharge	Usual outpatient physiotherapy care + activity tracker with weekly steps/day goal and monthly follow-up calls	10 weeks outpatient physiotherapy* + 6 months follow up	Yes (RL+OD)	NA	Fitbit Zip	Usual outpatient physiotherapy care	^a^**Steps/day** ^a^**(P, FU)** Minutes in moderate – vigorous PA	NA	NA	A PA intervention with supervision is feasible and may increase PA
Creel (2016) [55]	Patients after bariatric surgery	Intervention 1: n = 52, 8 (15), 42 ± 11 Intervention 2: n = 48, 8 (17), 44 ± 12 Control: n = 50, 8 (16), 44 ± 11	After hospital discharge	1)Pedometer intervention: Usual care + Pedometer + information sheet to increase PA to 10.000 steps/day 2)Counseling intervention: Usual care + Pedometer + exercise counseling with Motivational Interviewing (MI)	6 months	1)No 2)Yes (RL)	1)NA 2)Self-determination theory	Omron HJ 113 pedometer	Usual care	^a^**Steps/day** ^a^**(D, P)** % time spent in sedentary activity	^a^**Submaximal graded exercise test (P)**	NA	A counselling intervention using pedometers increased PA in the perioperative period
Dorsch (2015) [38]	Patients with stroke	Intervention: n = 78, 31 (40), 62 ± 16 Control: n = 73, 28 (38), 65 ± 13	During inpatient rehabilitation	Speed feedback + results and feedback on their summary activity graphs with a therapist	21 days*	Yes (RL)	NA	Tri-axial accelerometer (Gulf Coast Data Concepts)	Speed feedback only: verbal feedback about walking speed after 10m walking test.	^a^**Time spent walking (P)**	^a^**3-minute walking test (P)** 150m walking speed test	NA	Augmented feedback did not improve walking outcomes
Frederix (2015) [43]	Patients with acute coronary syndrome after a percutaneous coronary intervention or coronary artery bypass graft	Intervention: n = 32, 26 (81), 58 ± 9 Control: n = 34, 29 (85), 63 ± 10	During phase II cardiac rehabilitation.	Exercise training at home with telemonitoring support with accelerometers to encourage patients to increase his/her daily amount of steps wit 10% each week from baseline.	18 weeks	No	NA	Triaxial accelerometer (Yorbody company)	Exercise training in the hospital’s rehabilitation centre	NA	^a^**Maximal cardio-pulmonary exercise test (P)**	NA	PA monitoring might be effective to maintain exercise tolerance
Hassett (2020) [42]	Adults with mobility limitations undertaking aged care and neurological inpatient rehabilitation	Intervention: n = 149, 77 (52), 70 ± 18 Control: n = 151, 74 (49), 73 ± 15	During and after inpatient rehabilitation	Usual care + activity monitor, virtual reality video games and handheld computer devices with support by a physiotherapist	6 months	Yes (RL+OD)	NA	Fitbit Zip, One and Alta	Usual care	^a^**Steps/day (D,P)** Time spent walking/day % of the day spent upright	^a^**Short Physical Performance Battery (SPPB) (P)** DEMMI Step test	NA	The use of digitally enabled rehabilitation improved mobility
Hornikx (2015) [54]	Patients with COPD, hospitalized for an exacerbation of COPD	Intervention: n = 15, 8 (53), 66 ± 7 Control: n = 15, 9 (60), 68 ± 6	After hospital discharge	Pedometer + physical activity counselling with personalized goals	1 month	Yes (OD)	NA	Fitbit Ultra pedometer	Usual care	^a^**Steps/day (P)** Time spent walking/day	^a^**Six minutes walking test (P)** Quadriceps muscle strength	NA	PA counselling with pedometer feedback did not improve PA or clinical outcomes
Houle (2011) [44]	Patients < 80 years hospitalized for an acute coronary syndrome	Intervention: n = 32, 26 (81), 58 ± 8 Control: n = 33, 25 (76), 59 ± 9	After hospital discharge	Home based cardiac rehabilitation program + pedometer + exercise counseling by clinical nurse specialist with a target of 3000 steps per day increment in physical activity	12 months	Yes (RL+OD)	Social Cognitive theory	Yamax Digiwalker SW-200	Usual care	^a^**Steps/day** ^a^**(D, P)**	NA	NA	A pedometer intervention was useful to improve average steps/day
Izawa (2005) [37]	Patients after completion of an acute-phase inpatient cardiac rehabilitation program	Intervention: n = 24, 21 (88), 64 ± 10 Control: n = 21, 17 (81), 65 ± 10	After inpatient rehabilitation	Usual care + self-monitoring of physical activity with feedback from a physical therapist	5 months	Yes (RL)	Bandura’s self-efficacy theory	Kenz Liferecorder pedometer	Usual care	Steps/day (FU)	^a^**Cardio-pulmonary exercise test (P)** Hand grip strength Quadriceps muscle strength	NA	Self-monitoring of PA may effectively increase PA
Izawa (2012) [45]	Consecutive cardiovascular patients	Intervention: n = 52, 41 (79), 59 ± 8 Control: n = 51, 42 (82), 59 ± 13	During hospitalization until the first outpatient contact with a physician after discharge.	Usual care + self-monitoring of physical activity with feedback from a physical therapist	7 weeks*	Yes (RL)	Self-efficacy theory of Bandura and Oka	Kenz Lifecorder EX 1-axial accelerometer	Usual care	^a^**Steps/day (P)**	NA	NA	Self-monitoring of PA might effectively increase PA
Kanai (2018) [39]	Patients with acute ischemic stroke	Intervention: n = 23, 15 (65), 67 ± 10 Control: n = 25, 13 (52), 63 ± 9	During hospitalization	Usual care + self-monitoring of physical activity with feedback from a physical therapist	12 days*	Yes (RL)	Self-efficacy theory of Bandura	Fitbit One	Usual care	^a^**Steps/day (P)**	NA	NA	Exercise training with accelerometer-based feedback effectively increased PA
Lawrie (2018) [40]	Patients with recent stroke during rehabilitation	Intervention: n = 14, 10 (71), 53 ± 12 Control: n = 16, 13 (81), 62 ± 12	During inpatient rehabilitation	Usual care + smartwatch with visual feedback and a set goals based on a 5% increase in the total activity.	3 weeks*	No	NA	ZGPAX S8 Android smartwatch	Usual care + smartwatch with limited visual feedback without goal setting.	NA	^a^**Barthel Index (P)** 10m walk test Hand grip strength	NA	No effect was found on functional outcome
Mansfield (2015) [41]	Patients with sub-acute stroke attending inpatient rehabilitation	Intervention: n =29, 20 (69), 64 ± 19 Control: n = 28, 16 (57), 62 ± 13	During inpatient rehabilitation	Usual care + accelerometer-based daily walking activity reports with feedback from a physical therapist	2 weeks*	Yes (RL)	NA	Two tri-axial accelerometers (Gulf Data Concepts)	Usual care	^a^**Steps/day (P)** Time spent walking/day	^a^**6-meter walk test (P)**	NA	Feedback did not increase the amount of walking
Mehta (2020) [50]	Patients after hip or knee arthroplasty	Intervention: n=118, 38 (24), median age 66 (IQR 60-73) Control: n=124, 25 (20), median age 66 (IQR 57-73)	After hospital discharge	1) Intervention A: Usual care + remote monitoring alone 2)Intervention B: Usual care + remote monitoring with gamification and social support	45 days	1)No 2)No	NA	Withings physical activity monitor	Usual care	NA	^a^**Timed up and Go test (P)**	NA	PA monitoring did not improve functional outcomes
Moller (2015) [53]	Inactive patients with breast or colon cancer referred to adjuvant chemotherapy	Intervention: n = 14, 1 (7), 48 ± 8 Control: n = 16, 2 (13), 47 ± 9	After surgery, during adjuvant chemotherapy	Usual care + Home-based individual progressive pedometer intervention with health promotion counselling and symptom management by a clinical nurse specialist	12 weeks	Yes (RL)	NA	Omron Walking Style Pro pedometer	Usual care	NA	^a^**Cardio-respiratory fitness test (P)** Muscle strength (leg press and chest press)	NA	No effect was found on functional outcomes
Peel (2016) [56]	Patients admitted to post-acute care rehabilitation (aged 60 years and older)	Intervention: n = 128, 50 (39), 81 ± 9 Control: n = 127, 57 (45), 82 ± 8	During inpatient rehabilitation	Usual care + accelerometer based feedback and goal setting on daily walking time by therapist	4 weeks	Yes (RL)	NA	Triaxial ALIVE Heart and Activity Monitors and ActivPAL	Usual care	^a^**Non-therapy walking time** ^a^**(D, P)**	^a^**Short Physical Performance Battery (SPPB) (P)**	NA	Daily feedback on PA using accelerometers increased walking time
Pol (2019) [47]	Patients > 65 years old after hip fracture	Intervention: n =76, 11 (14), 84 ± 7 Control: n =87, 21 (24), 83 ± 7	During and after institutional-ization in a skilled nursing facility	Usual occupational care + Cognitive Behavioural Treatment (CBT) + sensor monitoring	4 months	Yes (RL)	Self-efficacy theory of Bandura	PAM AM300	Usual occupational care + CBT	NA	^a^**Performed-Oriented Mobility Assessment (POMA)** ^a^**(P, FU)** Timed up and Go test	^a^**Canadian Occupational Performance Measure (COPM) – performance scale (P, FU)**	Sensor monitoring occupation therapy was more effective in improving patient reported daily functioning than usual care
Van der Meij (2018) [51]	Adult patients scheduled for laparoscopic adnexal surgery, laparoscopic or open hernia inguinal surgery or laparoscopic cholecystectomy	Intervention: n = 173, 78 (45), 52 ± NA Control: n = 171, 79 (46), 51 ± NA	During and after hospitalization	Usual care + Personalized E-health program including self-monitoring on PA	6 weeks	Yes (OD)	NA	UP MOVE, Jawbone	Usual care	NA	NA	^a^**Patient Reported Outcomes Measurement Information System (PROMIS) – Physical Functioning** ^a^**(P)**	A personalised e-health program speeds up the return to normal activities compared to usual care
Van der Walt (2018) [48]	Adults undergoing primary elective hip or knee arthroplasty	Intervention: n = 81, 45 (56), 67 ± 9 Control: n = 82, 36 (44), 66 ± 9	During and after hospitalization	Usual care + activity tracker with daily step goals	6 weeks	No	NA	Garmin Vivofit 2	Usual care + activity tracker with obscured display	^a^**% of preoperative step count** ^a^**(D, P, FU)**	NA	Knee Injury and osteoarthritis outcome score (KOOS) (FU)	Patients who received feedback from a activity tracker had significant higher activity levels
Wolk (2019) [52]	Patients scheduled for elective open and laparoscopic surgery of the colon, rectum, stomach, pancreas or liver.	Intervention: n = 27, 16 (59), 61 ± 10 Control: n = 27, 19 (70), 56 ± 11.1	During the first 5 postoperative days	Usual care + activity trackers with daily step goals	5 days	No	NA	Polar Loop activity tracker	Usual care + activity tracker with obscured display	^a^**Steps/day (P)**			

*NA* not applicable, *dependent on admission time, *RL* real life, *OD* on distance, *D* during the intervention, *P* post-intervention, *FU* long term follow up, ^a^Bold = included in meta-analysis

### BCT coding

Overall, 20 of the 93 BCTs were coded exclusively in the intervention group compared to the control group. In two studies, two different interventions were included in the analyses; these interventions were coded on BCTs separately [50, 55]. Cohen’s kappa between both reviewers (ML & PB) was 0.93. One BCT was coded by the second reviewer, who checked 20% of the trials, which was not coded by the first reviewer. Therefore, all other trials were checked again for that specific BCT. Overall, an agreement between the reviewers was reached.

The amount of BCTs used in the included interventions ranged from 1 to 12, with a mean of 6.2 (SD = 2.96). The BCT feedback on behaviour was used in all interventions (*n* = 23). Other commonly used BCTs were goal setting (behaviour)(*n* = 15), action planning (*n* = 12), self-monitoring of behaviour (*n* = 15), graded tasks (*n* = 12) and adding objects to the environment (*n* = 15). An overview of the coded BCT per intervention is presented in Electronic Supplementary Material Table S2.

### Methodological quality

The results of the Risk of Bias assessment are presented in Table 2. Cohen’s kappa between both reviewers was 0.79 (ML & PB). After discussion, full consensus was reached between both reviewers. The PEDro score of the included trials ranged from 3 to 8. Thirteen trials were judged as low risk of bias and eight trials as high risk of bias. With the exception of one trial [55], all studies had clearly specified the eligibility criteria. The study of Brandes et al. (2018) performed a pseudo-randomization and was therefore negatively assessed on the randomization procedure. Blinding of participants and therapists was not possible in any study due to the intervention setting.

**Table 2:**
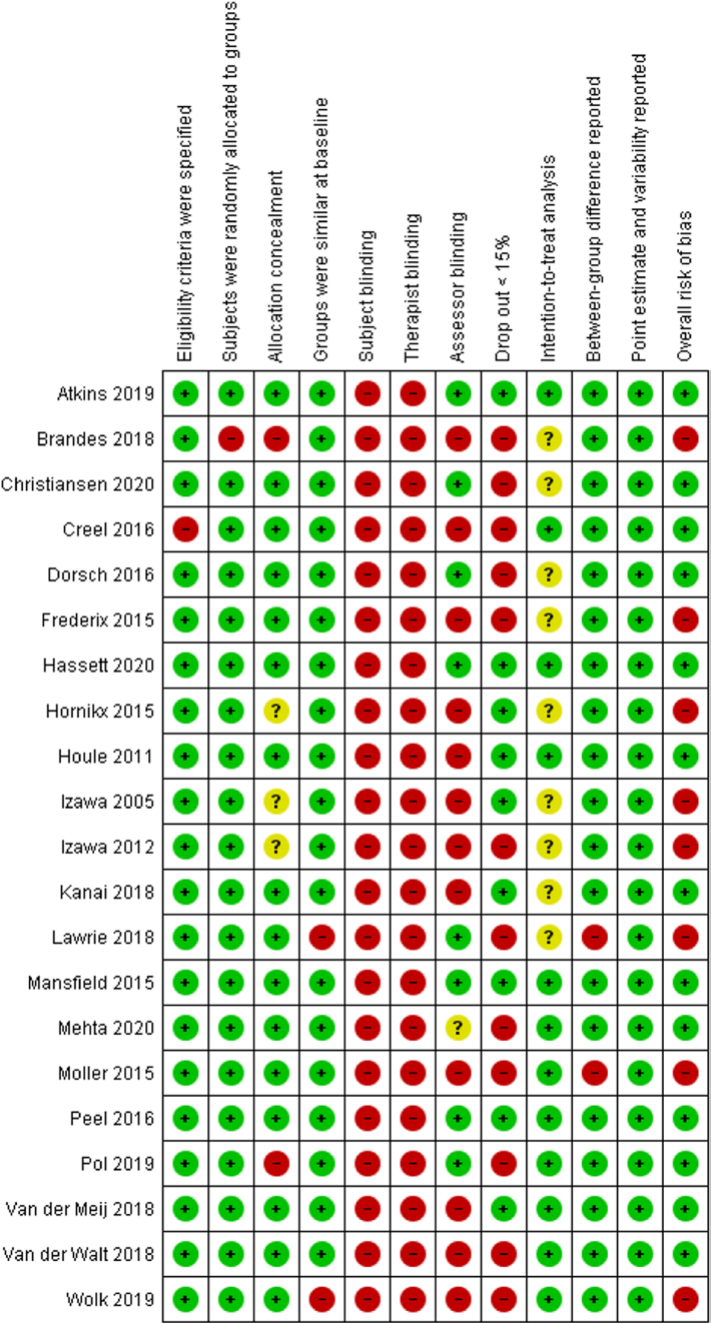
Risk of bias assessment of included studies (*n* = 21)

### Primary outcomes

#### Physical activity

Of the 21 included studies, 15 studies measured the effect of the intervention on objectively measured PA [37–39, 41, 42, 44–46, 48, 49, 52, 54–57]. The most frequent outcome measure of PA was steps per day, which was used in 11 studies [37, 39, 41, 42, 44–46, 49, 54, 55, 57]. Other outcome measures of PA were time spent walking [38], non-therapy walking time [56], percentage of preoperative step count at follow up [48] and mean step count during the first five postoperative days [52]. Six studies reported PA during the intervention of which five studies showed a significant positive effect in favour of the intervention group compared to the control group [48, 55–58]. The post-intervention outcome was reported in 13 studies; seven studies showed a significant positive effect in favour of the intervention group [39, 44, 45, 48, 49, 55, 56] and one study showed a significant positive effect in favour of the control group [52]. Four studies reported a long-term follow up of 6 months after intervention: three studies reported a significant positive effect in favour of the intervention group [37, 48, 49].

Meta-analysis was conducted for the mean difference between baseline and post-intervention comparing the intervention and control group, for which 13 studies provided data. Of these, only four studies reported the mean difference between baseline and post-intervention [38, 48, 52, 54], therefore the mean difference had to be calculated for the other studies. Three authors were contacted with success, because data to measure the mean difference was not available [48, 55, 56]. In the study of Creel et al. [55] and the study of Wolk et al. [52], data analysis was performed in two different population groups: these groups have been included separate in the meta-analysis.

**Fig. 2:**
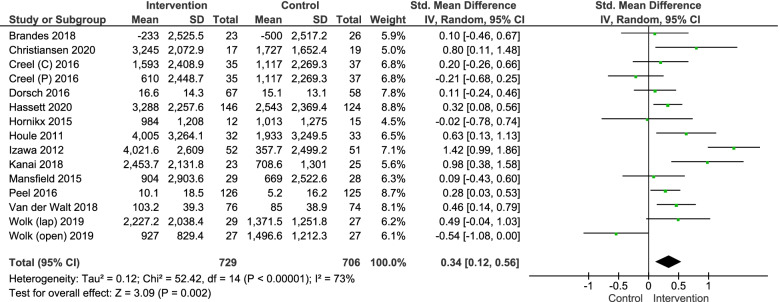
Forest plot for the outcome physical activity

Data was pooled in a random effects meta-analysis using data from 1435 participants (729 intervention/706 control). The model resulted in an overall estimated effect size in terms of standardized mean difference (SMD) of 0.34 (95%CI 0.12; 0.56) indicating a significant effect in favour of the intervention group (*p* = 0.002). The level of heterogeneity (I^2^) was 73% (Fig. 2). The Funnel plot is presented in Electronic Supplementary Material Fig. S1. Egger’s regression test indicated no significant asymmetry of the funnel plot (Egger’s Test = 0.205 *p* = 0.373). The SMD of Izawa et al. (2012) and Wolk et al. (open surgery) deviated the most from the overall effect size (SMD 1.42 and − 0.54, respectively). However, leave-one-out sensitivity analysis showed that the effect sizes remained within the 95%CI after iteratively removing both studies from analysis (SMD 0.26, 95%CI 0.09;0.43, *p* = 0.003 resp. SMD 0.40, 95%CI 0.19; 0.60, *p* = 0.0002).

#### Physical functioning (performance based)

A total of 13 trials reported a performance based outcome of physical functioning [37, 38, 40–43, 47, 50, 53–57]. The most common used outcome measure was peak oxygen uptake (peak VO_2_) measured during an cardiopulmonary exercise test and was reported in three studies [37, 43, 53]. Other outcome measures were the Short Physical Performance Battery [42, 56], 3 or 3 min walking distance [38, 54], the Morton Mobility Index [57], exercise tolerance (MET’s) [55], the Barthel Index [40], walking speed [41], the Performance-Oriented Mobility Assessment [47] and the Timed Up and Go test [50]. All studies reported post-intervention outcome of which three reported a significant positive effect in favour of the intervention group [41–43]. Only the study of Pol et al. reported a long term follow-up, but did not found a significant effect [47].

**Fig. 3:**
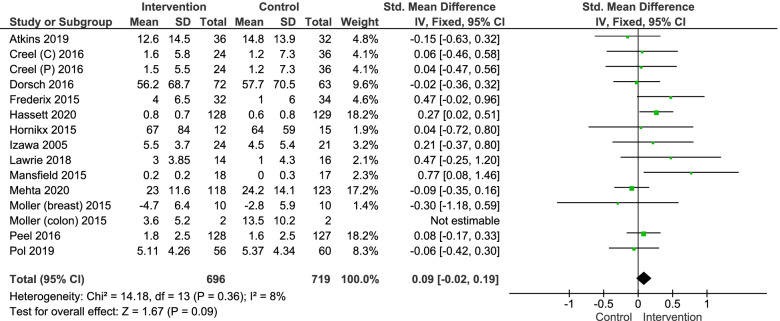
Forest plot for outcome performance based physical functioning

The mean difference was reported in two studies and had to be calculated for the other ten studies. The study of Creel et al. [55] included two different intervention groups (see Table 1), therefore these groups have been included separate in the meta-analysis. In the study of Moller et al. [53] data analysis was performed in two different population groups (colon and breast cancer). However, one group has been excluded for meta-analysis due to the low number of participants in both intervention and control group (*n* = 4). In the study of Mehta et al., only the median and IQR were reported, therefore the sample mean and SD were estimated as described in the method section. Data was pooled in a random effects model meta-analysis including 1415 participants (696 intervention/719 control). The model resulted in an overall estimated effect size in terms of standardized mean difference of 0.09 (95%CI -0.02; 0.20, I^2^ = 8%). No significant effect was found between groups (*P* = 0.11) (Fig. 3). Funnel plot (Electronic Supplementary Material Fig. S2) and Egger’s regression test indicated that publication bias was unlikely to have influenced de results (Egger’s Test = − 0,063; *p* = 0.914).

#### Physical functioning (patient reported)

Four studies reported a PROM of physical functioning [46–48, 51]. The study of Brandes et al. used the Oxford knee/hip score as outcome, but did not find a significant difference between the intervention and control group post-intervention or at 6 months follow up [46]. Also in the study of Van der Walt et al., no significant effect was found at 6 months follow up on the Knee Injury and Osteoarthritis Outcome Score [48]. On the other hand, a significant positive treatment effect was found on the Canadian Occupational Performance Measure (COPM) post intervention and at 6 months follow-up in the study of Pol et al. [47] In the study of van der Meij et al., a significant positive effect on the median days return to normal activities, measured with the Patient Reported Outcomes Measurement Information System – Physical Functioning (PROMIS-PF), was found in favour of the intervention group [51]. However, no significant difference between groups was found in the PROMIS-PF post-intervention compared to baseline.

**Fig. 4:**
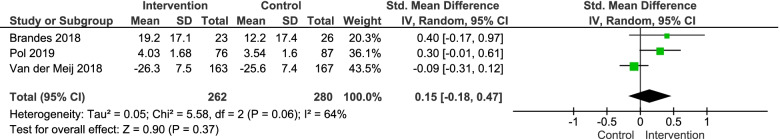
Forest plot for patient reported outcome measure of physical functioning

Overall, meta-analysis of patient reported outcome of physical functioning post-intervention using a random effects model resulted in an overall estimated effect size of 0.15 (95% CI -0.18; 0.47) (Fig. 4). A funnel plot and Eggers test was not performed because of the low number of included studies.

### Subgroup analysis study characteristics

Meta-analysis for PA presented high heterogeneity (73%, see Fig. 2), therefore subgroup analyses were conducted to explore the contribution of different study characteristics on the overall effect. No significant differences were found between subgroups (Table 3). However, interventions that took place both during and after the inpatient period showed a high significant effect in favour of the intervention group (SMD = 0.71, 95%CI 0.13;1.29), whereas interventions that only took place during or after the inpatient period did not reveal significant effects (SMD = 0.21, 95%CI -0.07; 0.48 resp. SMD = 0.26, 95%CI -0.11; 0.64). This also applies for the age group, however differences in effect sizes were less in these groups (Table 3). Methodological quality had no significant effect on effect size (*p* = 0.97): studies with a higher risk of bias did not result in different effect sizes.

**Table 3:** Subgroup analysis study characteristics

Study characteristics	Outcome PA (*n* = 15)				
	n	Combined sample size	Pooled mean SMD (95% CI)	Q	p
**Setting**				0.88	0.35
Hospitalization	10	683	0.43 (0.06; 0.79)*		
Rehabilitation	5	752	0.24 (0.10; 0.38)*		
**Period**				2.38	0.30
During	7	640	0.21 (−0.07; 0.48)		
After	5	272	0.26 (−0.11; 0.64)		
During and after	3	523	0.71 (0.13; 1.29)*		
**Duration of the intervention**				0.04	0.84
≤3 months	10	920	0.35 (0.04; 0.66)*		
> 3 months	5	515	0.31 (0.02; 0.60)*		
**Age group**				0.00	0.95
Mean age ≤ 60 years	6	422	0.34 (−0.23; 0.91)		
Mean age > 60 years	9	1013	0.32 (0.16; 0.48)*		
**Risk of Bias**				0.00	0.97
Low risk	10	1146	0.32 (0.15; 0.49)*		
High risk	5	289	0.31 (−0.42; 1.04)		

*p < 0.05, Q = cochrane’s Q

Subgroup analysis for the outcome performance based and patient reported physical functioning were not conducted, because the meta-analysis either presented low heterogeneity (I^2^ = 8%) or included a low number of studies.

### Subgroup analysis intervention characteristics

The mean number of BCTs in the included interventions was 6.4. Therefore, subgroup analysis was conducted for interventions with ≥7 BCTs and < 7 BCTs. Interventions with ≥7 BCTs showed an significant effect on PA (SMD = 0.60, 95%CI 0.18;1.02, *p* = 0.005), whereas interventions with < 7 BCTs did not (SMD = 0.18, 95%CI -0.04;0.39, *p* = 0.11). The forest plot is presented in Electronic Supplementary Material Fig. S3. The following BCTs were only used in the subgroup with ≥7 BCTs: problem solving (*n* = 5), instructions on how to perform a behavior (*n* = 3), information about health consequences (*n* = 1), information about social and environmental consequences (*n* = 1), social comparison (*n* = 1), prompts/cues (*n* = 3) and social reward (*n* = 2).

The SMD of theory-based interventions with activity trackers was higher (SMD = 0.66, 95%CI 0.14; 1.18, *p* = 0.01) compared to interventions without a theoretical model (SMD = 0.20. 95%CI -0.00; 0.40, *p* = 0.04)(Electronic Supplementary Material Fig. S4). The mean number of BCTs used in theory-based interventions was higher: 8.4 compared to 5.3. The BCTs that were exclusively coded in the subgroup with theory-based interventions were: information about health consequences (*n* = 1), information about social and environmental consequences (*n* = 1), social comparison (*n* = 1) and social reward (*n* = 2).

Interventions with coaching by a health professional showed a larger effect on PA (SMD = 0.44, 95%CI 0.19; 0.69, *p* = 0.0004) compared to interventions without coaching by a health professional (SMD = 0.07, 95%CI -0.42; 0.56, *p* = 0.78) (Electronic Supplementary Material Fig. S5). In the interventions with supervision by a health professional more different BCTs were used: the mean number of BCTs was 6.8 compared to 4.8. The following BCTs were exclusively coded in interventions with coaching by a health professional: problem solving (*n* = 5), review behaviour goals (*n* = 4), instructions on how to perform a behaviour (*n* = 3), information about health consequences (*n* = 1), information about social and environmental consequences (*n* = 1), social comparison (*n* = 1), prompts/cues (*n* = 3) and social reward (*n* = 3).

## Discussion

The results of this systematic review and meta-analysis showed that interventions using activity trackers during and/or after inpatient care are heterogeneous, but are generally more effective in increasing the level of PA compared to usual care. However, this does not necessarily translate into an improvement in physical functioning. There was high variability of study populations, characteristics and intervention strategies across the included studies. Subgroup analysis of study characteristics suggest that interventions taking place both during and after an inpatient period may be more effective in stimulating PA compared to interventions only during or only after inpatient treatment. In addition, interventions using more BCTs, theory based interventions and interventions in combination with coaching by a health professional also seem to increase the effect on the level of PA.

A small positive effect on PA in favour of the intervention group was found. These results are in line with the results of meta-analyses in other patient populations [15–18]. In the review of Braakhuis et al., a small positive effect of healthcare interventions using objective feedback on PA was found (SMD = 0.34, *p* < 0.01) in a heterogeneous patient population (patients with COPD, stroke, cardio-vascular diseases, Parkinson’s disease and geriatric patients) [18]. A moderate positive effect on PA was found in a meta-analysis in people with type 2 diabetes (SMD 0.57, p < 0.01) and in a meta-analysis in patients with COPD using step counters (SMD 0.57, *p* < 0.05) [15, 17]. A high positive effect on daily step count was found in a meta-analysis in patients with rheumatic and musculoskeletal diseases (SMD 0.83, p < 0.01) [16]. The lower effect in our study compared to these studies may be caused by patients experiencing more barriers to increase their level of PA during or after inpatient care due to impact of the ‘acute event’ (e.g. having symptoms, such as pain or fatigue or due to overall reduced strength and condition as result of the acute event) compared to patients with chronic conditions in a daily life setting [10, 11, 13].

Although a positive effect was found on PA in favour of the intervention group, no effect was found on the outcome physical functioning in our meta-analysis. In other patient populations, previous reviews have found conflicting results on the effectiveness of activity tracker interventions on physical functioning. A small significant positive effect was found on physical functioning in patients with COPD (SMD = 0.32, *p* < 0.05) [17], whereas no significant effect was found in patients with rheumatic and musculoskeletal diseases (SMD = 0.09, *p* > 0.05) [16]. Of the individual included studies in our meta-analysis, two studies supported the effect that increased PA contributes to recovery in physical functioning [42, 43]. In contrast, no significant effect on physical functioning was found in four studies reporting a significant effect on PA in favour of the intervention group [48, 55–57]. One possible explanation for these differences in effectiveness is the timing of physical functioning measurements, as PA-interventions may have more effect on the rate than on the level of functional recovery. In other words, patients in the intervention group may have a physical functioning level similar to that of the control group after a certain time, but it may take them less time to reach that level. This could be particularly true in patient populations that fully recover to their pre-treatment physical functioning levels. Another explanation could be the very low number of studies that used a patient-reported outcome measure of physical functioning. Patient-reported outcomes are important because they can provide unique information on the impact of a medical condition and its intervention from the patient’s perspective. Finally, high variability in outcome measures and small sample sizes in our review lead to low certainty of evidence of the results for both performance-based and patient-reported physical functioning, according to the Grading of Recommendations Assessment, Development and Evaluation (GRADE) approach [59]. To gain a better understanding of the effect of interventions using activity trackers during and/or after inpatient care, conducting clinical trials measuring both patient-reported and performance-based outcomes of physical functioning at multiple follow-up times is warranted.

Subgroup analysis of study characteristics suggested that interventions conducted both during and after an inpatient period may be more effective in increasing the level of PA. This may be explained by the fact that in the interventions during the inpatient period, activity trackers were often added to standard interventions aimed at improving PA, whereas in the interventions after discharge, the activity tracker was often the only component aimed at improving PA. Three studies that conducted the intervention only during inpatient rehabilitation also mentioned the high load of usual rehabilitation care in the control group as possible explanation that no significant effect on PA was found [38, 41, 46]. In most cases priority was even given in the intervention group to the rehabilitation goals of usual care instead of the experimental intervention goals (daily step count). Also, if the intervention starts during inpatient stay and continues after discharge, patients might be more aware of their PA behaviour being back at home. Therefore, it is suggested that these interventions may be more effective when implemented both during and after inpatient care.

Our results support previous studies suggesting that theory-based interventions are generally more effective in promoting PA [23–26]. It is assumed that in theory-based interventions, the active ingredients of the interventions are more carefully described and implemented. This is supported by our results of coded BCTs in both subgroups, as the mean number of coded BCTs was higher in theory-based interventions (8.4 vs. 5.7).

Interventions using a higher number of BCTs were found to be more effective in improving PA, as also found in other studies [60, 61]. This is in line with the finding that interventions with coaching by a health professional are more effective, because more different BCTs can be used if interventions are supported by a health professional (e.g. problem solving, social reward). Besides that, it is suggested that activity trackers as standalone intervention might not be sufficient for special patient populations, because most activity trackers do not include BCTs that are specific to a certain population [22, 62]. Incorporating coaching by a health professional to the intervention gives the opportunity to provide targeted advice and interventions for a specific population group with a more personal touch. These findings are also supported by earlier research [27, 63].

Results suggest that interventions using activity trackers increase PA levels of surgical and non-surgical patients during and/or after inpatient care. The advantage of activity trackers is the minimal burden on the user in relation to the data that can be produced, and the ability to provide real-time feedback on PA. Activity trackers can thereby motivate and support patients and reduce the time and resources required for traditional methods of ongoing support.

### Strengths and limitations

To our knowledge, this is the first meta-analysis investigating the effect of interventions with activity trackers in patients during and/or after inpatient care. The study provides insight into which intervention characteristics may improve the effectiveness, which can be helpful in the development of interventions with activity trackers in this population. An internationally validated taxonomy was used to identify BCTs in these interventions. Two trained researchers coded BCTs individually and agreement was received through discussion. Other strengths of this study are that only objective data of PA was used as outcome measurement for PA and that the outcome measurements were corrected for baseline status.

This study has also several limitations. First, there was considerable heterogeneity among the included studies in terms of study populations, duration of intervention and intensity of intervention. Because the high level of heterogeneity, standardized mean difference (SMD) was used. However, using SMD only partly resolves the problem of comparing different outcomes. Therefore, results should be interpreted carefully. Second, heterogeneity in the terminology and insufficient description of the active ingredients of the interventions impaired the coding of BCTs. As a result, it is likely that BCTs are underreported. Unfortunately, this problem is common in research on the effect of different BCTs [64]. Third, not all studies reported the mean difference between the post-intervention and baseline measurement. In these studies, the mean difference was calculated based on available or requested data. In the study of Hassett et al., this has led to a difference in significance of the outcome due to a different analysis method [42]. Our calculation of the mean difference in the study of Hassett et al. resulted in a significant effect on PA, whereas Hassett et al. reported a non-significance effect (*p* = 0.09). However, the estimated effect was roughly similar to our result. Finally, the meta-analysis could only be conducted for short-term outcomes (post-intervention), due to the lack of long-term outcomes (e.g., 3 or 6 months of follow-up). However, it is likely that the effect of interventions and the role of BCTs differ between short- and long-term outcome assessments [65], so intervention studies are encouraged to include long-term outcome assessments.

## Conclusion

Interventions using activity trackers during and/or after inpatient care have the potential to increase the level of PA across a wide range of surgical and non-surgical populations. Despite the expectation that higher levels of PA have a positive effect on physical functioning, no significant effect on physical functioning was found. The intensity and quality of the interventions seem to improve by providing the intervention both during and after the inpatient period, by using more BCTs, integrating a theoretical model, and providing coaching by a healthcare professional, as a greater effect on PA increase has been found in studies using these intervention characteristics. Thus, interventions using activity trackers have the potential to be included as an effective tool to motivate patients and to assist health professionals to provide ongoing monitoring and support with minimal resource expenditure. However, results of this review should be interpreted carefully due to the high heterogeneity between studies. Future RCTs investigating the use of activity trackers should investigate the effect on the course of recovery in physical functioning and should pay attention to a sufficient description of the active ingredients of both the intervention and control conditions, enabling the comparison of different BCTs on outcomes of these interventions.

## Supplementary Information

**Additional file 1.**

**Additional file 2.**

## Data Availability

The datasets generated during and analysed during the current study are available from the corresponding author on reasonable request.

## Abbreviations

BCT: Behavioral change technique
IQR: Interquartile range
PA: Physical activity
RCT: Randomized Controlled Trial
SD: Standard Deviation
SMD: Standardized Mean Difference

## References

[1] Cabilan CJ, Hines S. The short-term impact of colorectal cancer treatment on physical activity, functional status and quality of life: a systematic review. JBI Database System Rev Implement Rep. 2017;15(2). 10.11124/jbisrir-2016003282. 10.11124/JBISRIR-2016003282 28178025

[2] van Zutphen M, Winkels RM, van Duijnhoven FJ, van Harten-Gerritsen SA, Kok DE, van Duijvendijk P, et al. An increase in physical activity after colorectal cancer surgery is associated with improved recovery of physical functioning: a prospective cohort study. BMC Cancer. 2017;17(1). 10.1186/s12885-017-3066-2. 10.1186/s12885-017-3066-2 PMC5264442 28122534

[3] Lawrence VA, Hazuda HP, Cornell JE, Pederson T, Bradshaw PT, Mulrow CD, et al. Functional independence after major abdominal surgery in the elderly. J Am Coll Surg. 2004;199(5). 10.1016/j.jamcollsurg.2004.05.280. 10.1016/j.jamcollsurg.2004.05.280 15501119

[4] Hara T, Kubo A. Relationship between physical activity and function in elderly patients discharged after surgical treatment for gastrointestinal cancer. J Phys Ther Sci. 2015;27(9). 10.1589/jpts.27.2931. 10.1589/jpts.27.2931 PMC4616128 26504327

[5] Askim T, Bernhardt J, Churilov L, Fredriksen KR, Indredavik B. Changes in physical activity and related functional and disability levels in the first six months after stroke: a longitudinal follow-up study. J Rehabil Med. 2013;45(5). 10.2340/16501977-1137. 10.2340/16501977-1137 23571658

[6] van der Leeden M, Balland C, Geleijn E, Huijsmans RJ, Dekker J, Paul MA, et al. In-hospital mobilization, physical fitness, and physical functioning after lung Cancer surgery. Ann Thorac Surg. 2019;107(6). 10.1016/j.athoracsur.2018.12.045. 10.1016/j.athoracsur.2018.12.045 30690020

[7] Hokstad A, Indredavik B, Bernhardt J, Langhammer B, Gunnes M, Lundemo C, et al. Upright activity within the first week after stroke is associated with better functional outcome and health-related quality of life: a Norwegian multi-site study. J Rehabil Med. 2016;48(3). 10.2340/16501977-2051. 10.2340/16501977-2051 26843147

[8] Resnick B, Boltz M. Optimizing function and physical activity in hospitalized older adults to prevent functional decline and falls. Clin Geriatr Med. 2019;35(2). 10.1016/j.cger.2019.01.003. 10.1016/j.cger.2019.01.003 30929885

[9] Brown CJ, Williams BR, Woodby LL, Davis LL, Allman RM. Barriers to mobility during hospitalization from the perspectives of older patients and their nurses and physicians. J Hosp Med. 2007;2(5). 10.1002/jhm.209. 10.1002/jhm.209 17935241

[10] Hoyer EH, Brotman DJ, Chan KS, Needham DM. Barriers to early mobility of hospitalized general medicine patients: survey development and results. Am J Phys Med Rehabil. 2015;94(4). 10.1097/phm.0000000000000185. 10.1097/PHM.0000000000000185 PMC4344426 25133615

[11] Granger CL, Connolly B, Denehy L, Hart N, Antippa P, Lin KY, et al. Understanding factors influencing physical activity and exercise in lung cancer: a systematic review. Support Care Cancer. 2017;25(3). 10.1007/s00520-016-3484-8. 10.1007/s00520-016-3484-8 27900549

[12] Geelen SJG, Giele BM, Engelbert RHH, de Moree S, Veenhof C, Nollet F, et al. Barriers to and solutions for improving physical activity in adults during hospital stay: a mixed-methods study among healthcare professionals. Disabil Rehabil. 2021. 10.1080/09638288.2021.1879946. 10.1080/09638288.2021.1879946 33605171

[13] Koenders N, Weenk M, van de Belt TH, van Goor H, Hoogeboom TJ, Bredie SJH. Exploring barriers to physical activity of patients at the internal medicine and surgical wards: a retrospective analysis of continuously collected data. Disabil Rehabil. 2019. 10.1080/09638288.2019.1685013. 10.1080/09638288.2019.1685013 31691603

[14] de Vries HJ, Kooiman TJ, van Ittersum MW, van Brussel M, de Groot M. Do activity monitors increase physical activity in adults with overweight or obesity? A systematic review and meta-analysis. Obesity (Silver Spring). 2016;24(10). 10.1002/oby.21619. 10.1002/oby.21619 27670401

[15] Baskerville R, Ricci-Cabello I, Roberts N, Farmer A. Impact of accelerometer and pedometer use on physical activity and glycaemic control in people with type 2 diabetes: a systematic review and meta-analysis. Diabet Med. 2017;34(5). 10.1111/dme.13331. 10.1111/dme.13331 28173623

[16] Davergne T, Pallot A, Dechartres A, Fautrel B, Gossec L. Use of wearable activity trackers to improve physical activity behavior in patients with rheumatic and musculoskeletal diseases. A Systematic Review and Meta-Analysis Arthritis Care Res (Hoboken). 2019;71(6). 10.1002/acr.23752. 10.1002/acr.23752 30221489

[17] Qiu S, Cai X, Wang X, He C, Zugel M, Steinacker JM, et al. Using step counters to promote physical activity and exercise capacity in patients with chronic obstructive pulmonary disease: a meta-analysis. Ther Adv Respir Dis. 2018;12. 10.1177/1753466618787386. 10.1177/1753466618787386 PMC6048621 29993339

[18] Braakhuis HEM, Berger MAM, Bussmann JBJ. Effectiveness of healthcare interventions using objective feedback on physical activity: a systematic review and meta-analysis. J Rehabil Med. 2019;51(3). 10.2340/16501977-2522. 10.2340/16501977-2522 30843082

[19] Bell K. Remaking the self: trauma, teachable moments, and the biopolitics of cancer survivorship. Cult Med Psychiatry. 2012;36(4). 10.1007/s11013-012-9276-9. 10.1007/s11013-012-9276-9 PMC3496522 23054293

[20] Robinson A, Slight R, Husband A, Slight S. The value of teachable moments in surgical patient care and the supportive role of digital technologies. Perioper Med (Lond). 2020;9. 10.1186/s13741-019-0133-z. 10.1186/s13741-019-0133-z PMC6998815 32042404

[21] Michie S, Richardson M, Johnston M, Abraham C, Francis J, Hardeman W, et al. The behavior change technique taxonomy (v1) of 93 hierarchically clustered techniques: building an international consensus for the reporting of behavior change interventions. Ann Behav Med. 2013;46(1). 10.1007/s12160-013-9486-6. 10.1007/s12160-013-9486-6 23512568

[22] Lyons EJ, Lewis ZH, Mayrsohn BG, Rowland JL. Behavior change techniques implemented in electronic lifestyle activity monitors: a systematic content analysis. J Med Internet Res. 2014;16(8). 10.2196/jmir.3469. 10.2196/jmir.3469 PMC4147713 25131661

[23] Webb TL, Joseph J, Yardley L, Michie S. Using the internet to promote health behavior change: a systematic review and meta-analysis of the impact of theoretical basis, use of behavior change techniques, and mode of delivery on efficacy. J Med Internet Res. 2010;12(1). 10.2196/jmir.1376. 10.2196/jmir.1376 PMC2836773 20164043

[24] Gourlan M, Bernard P, Bortolon C, Romain AJ, Lareyre O, Carayol M, et al. Efficacy of theory-based interventions to promote physical activity. A meta-analysis of randomised controlled trials. Health. Psychol Rev. 2016;10(1). 10.1080/17437199.2014.981777. 10.1080/17437199.2014.981777 25402606

[25] Avery L, Flynn D, Dombrowski SU, van Wersch A, Sniehotta FF, Trenell MI. Successful behavioural strategies to increase physical activity and improve glucose control in adults with type 2 diabetes. Diabet Med. 2015;32(8). 10.1111/dme.12738. 10.1111/dme.12738 PMC6680111 25764343

[26] Finne E, Glausch M, Exner AK, Sauzet O, Stölzel F, Seidel N. Behavior change techniques for increasing physical activity in cancer survivors: a systematic review and meta-analysis of randomized controlled trials. Cancer Manag Res. 2018;10. 10.2147/cmar.S170064. 10.2147/CMAR.S170064 PMC6215922 30464612

[27] Spring B, Stump T, Penedo F, Pfammatter AF, Robinson JK. Toward a health-promoting system for cancer survivors: patient and provider multiple behavior change. Health Psychol. 2019;38(9). 10.1037/hea0000760. 10.1037/hea0000760 PMC6709684 31436465

[28] Page MJ, McKenzie JE, Bossuyt PM, Boutron I, Hoffmann TC, Mulrow CD, et al. The PRISMA 2020 Statement: an updated guideline for reporting systematic reviews. BMJ (Clinical research ed) 2021;372 doi: http://dx.doi.org/10.1136/bmj.n71. 10.1136/bmj.n71 PMC8005924 33782057

[29] World Health Organization. Physical Activity: World Health Organization; 2020 [Available from: https://www.who.int/news-room/fact-sheets/detail/physical-activity.

[30] Garber CE, Greaney ML, Riebe D, Nigg CR, Burbank PA, Clark PG. Physical and mental health-related correlates of physical function in community dwelling older adults: a cross sectional study. BMC Geriatr. 2010;10. 10.1186/1471-2318-10-6. 10.1186/1471-2318-10-6 PMC2835714 20128902

[31] de Morton NA. The PEDro scale is a valid measure of the methodological quality of clinical trials: a demographic study. Aust J Physiother. 2009;55(2). 10.1016/s0004-9514(09)70043-1. 10.1016/s0004-9514(09)70043-1 19463084

[32] Maher CG, Sherrington C, Herbert RD, Moseley AM, Elkins M. Reliability of the PEDro scale for rating quality of randomized controlled trials. Phys Ther. 2003;83(8). 12882612

[33] Cashin AG, McAuley JH. Clinimetrics: Physiotherapy evidence database (PEDro) scale. J Physiother 2020;66(1) doi: 10.1016/j.jphys.2019.08.005. 10.1016/j.jphys.2019.08.005 31521549

[34] Wan X, Wang W, Liu J, Tong T. Estimating the sample mean and standard deviation from the sample size, median, range and/or interquartile range. BMC Med Res Methodol. 2014;14. 10.1186/1471-2288-14-135. 10.1186/1471-2288-14-135 PMC4383202 25524443

[35] Cohen J (1988). Statistical power analysis for the behavioral sciences.

[36] Michie S, Abraham C, Whittington C, McAteer J, Gupta S. Effective techniques in healthy eating and physical activity interventions: a meta-regression. Health Psychol. 2009;28(6). 10.1037/a0016136. 10.1037/a0016136 19916637

[37] Izawa KP, Watanabe S, Omiya K, Hirano Y, Oka K, Osada N, et al. Effect of the self-monitoring approach on exercise maintenance during cardiac rehabilitation: a randomized, controlled trial. Am J Phys Med Rehabil. 2005;84(5). 10.1097/01.phm.0000156901.95289.09. 10.1097/01.phm.0000156901.95289.09 15829777

[38] Dorsch AK, Thomas S, Xu X, Kaiser W, Dobkin BH. SIRRACT: An international randomized clinical trial of activity feedback during inpatient stroke rehabilitation enabled by wireless sensing. Neurorehabil Neural Repair 2015;29(5) doi: 10.1177/1545968314550369. 10.1177/1545968314550369 PMC4375021 25261154

[39] Kanai M, Izawa KP, Kobayashi M, Onishi A, Kubo H, Nozoe M, et al. Effect of accelerometer-based feedback on physical activity in hospitalized patients with ischemic stroke: a randomized controlled trial. Clin Rehabil. 2018;32(8). 10.1177/0269215518755841. 10.1177/0269215518755841 29400070

[40] Lawrie S, Dong Y, Steins D, Xia Z, Esser P, Sun S, et al. Evaluation of a smartwatch-based intervention providing feedback of daily activity within a research-naive stroke ward: a pilot randomised controlled trial. Pilot Feasibility Stud. 2018;4. 10.1186/s40814-018-0345-x. 10.1186/s40814-018-0345-x PMC6173888 30323946

[41] Mansfield A, Wong JS, Bryce J, Brunton K, Inness EL, Knorr S, et al. Use of accelerometer-based feedback of walking activity for appraising Progress with walking-related goals in inpatient stroke rehabilitation: a randomized controlled trial. Neurorehabil Neural Repair. 2015;29(9). 10.1177/1545968314567968. 10.1177/1545968314567968 25605632

[42] Hassett L, van den Berg M, Lindley RI, Crotty M, McCluskey A, van der Ploeg HP, et al. Digitally enabled aged care and neurological rehabilitation to enhance outcomes with activity and MObility UsiNg technology (AMOUNT) in Australia: a randomised controlled trial. PLoS Med. 2020;17(2). 10.1371/journal.pmed.1003029. 10.1371/journal.pmed.1003029 PMC7028259 32069288

[43] Frederix I, Van Driessche N, Hansen D, Berger J, Bonne K, Alders T, et al. Increasing the medium-term clinical benefits of hospital-based cardiac rehabilitation by physical activity telemonitoring in coronary artery disease patients. Eur. J Prev Cardiol. 2015;22(2). 10.1177/2047487313514018. 10.1177/2047487313514018 24249840

[44] Houle J, Doyon O, Vadeboncoeur N, Turbide G, Diaz A, Poirier P. Innovative program to increase physical activity following an acute coronary syndrome: randomized controlled trial. Patient Educ Couns. 2011;85(3). 10.1016/j.pec.2011.03.018. 10.1016/j.pec.2011.03.018 21546203

[45] Izawa KP, Watanabe S, Hiraki K, Morio Y, Kasahara Y, Takeichi N, et al. Determination of the effectiveness of accelerometer use in the promotion of physical activity in cardiac patients: a randomized controlled trial. Arch Physical Med Rehabil 2012;93(11) doi: 10.1016/j.apmr.2012.06.015. 10.1016/j.apmr.2012.06.015 22750166

[46] Brandes M, Wirsik N, Niehoff H, Heimsoth J, Mohring B. Impact of a tailored activity counselling intervention during inpatient rehabilitation after knee and hip arthroplasty - an explorative RCT. BMC Musculoskelet Disord. 2018;19(1). 10.1186/s12891-018-2130-7. 10.1186/s12891-018-2130-7 PMC6026519 29960605

[47] Pol MC, Ter Riet G, van Hartingsveldt M, Krose B, Buurman BM. Effectiveness of sensor monitoring in a rehabilitation programme for older patients after hip fracture: a three-arm stepped wedge randomised trial. Age Ageing. 2019;48(5). 10.1093/ageing/afz074. 10.1093/ageing/afz074 31204776

[48] Van der Walt N, Salmon LJ, Gooden B, Lyons MC, O'Sullivan M, Martina K, et al. Feedback from activity trackers improves daily step count after knee and hip Arthroplasty: a randomized controlled trial. J Arthroplast. 2018;33(11). 10.1016/j.arth.2018.06.024. 10.1016/j.arth.2018.06.024 30017217

[49] Christiansen MB, Thoma LM, Master H, Voinier D, Schmitt LA, Ziegler ML, et al. Feasibility and preliminary outcomes of a physical therapist-administered physical activity intervention after Total knee replacement. Arthritis Care Res (Hoboken). 2020;72(5). 10.1002/acr.23882. 10.1002/acr.23882 PMC6761055 30908867

[50] Mehta SJ, Hume E, Troxel AB, Reitz C, Norton L, Lacko H, et al. Effect of remote monitoring on discharge to home, return to activity, and Rehospitalization after hip and knee Arthroplasty: a randomized clinical trial. JAMA Netw Open. 2020;3(12). 10.1001/jamanetworkopen.2020.28328. 10.1001/jamanetworkopen.2020.28328 PMC7753899 33346847

[51] van der Meij E, Anema JR, Leclercq WKG, Bongers MY, Consten ECJ, Schraffordt Koops SE, et al. Personalised perioperative care by e-health after intermediate-grade abdominal surgery: a multicentre, single-blind, randomised, placebo-controlled trial. Lancet (London, England). 2018;392(10141). 10.1016/s0140-6736(18)31113-9. 10.1016/S0140-6736(18)31113-9 29937195

[52] Wolk S, Linke S, Bogner A, Sturm D, Meissner T, Mussle B, et al. Use of activity tracking in major visceral surgery-the enhanced perioperative mobilization trial: a randomized controlled trial. J Gastrointest Surg. 2019;23(6). 10.1007/s11605-018-3998-0. 10.1007/s11605-018-3998-0 30298422

[53] Moller T, Lillelund C, Andersen C, Bloomquist K, Christensen KB, Ejlertsen B, et al. The challenge of preserving cardiorespiratory fitness in physically inactive patients with colon or breast cancer during adjuvant chemotherapy: a randomised feasibility study. BMJ Open Sport Exerc Med. 2015;1(1). 10.1136/bmjsem-2015-000021. 10.1136/bmjsem-2015-000021 PMC5117008 27900123

[54] Hornikx M, Demeyer H, Camillo CA, Janssens W, Troosters T. The effects of a physical activity counseling program after an exacerbation in patients with chronic obstructive pulmonary disease: a randomized controlled pilot study. BMC Pulm Med. 2015;15. 10.1186/s12890-015-0126-8. 10.1186/s12890-015-0126-8 PMC4632467 26530543

[55] Creel DB, Schuh LM, Reed CA, Gomez AR, Hurst LA, Stote J, et al. A randomized trial comparing two interventions to increase physical activity among patients undergoing bariatric surgery. Obesity (Silver Spring). 2016;24(8). 10.1002/oby.21548. 10.1002/oby.21548 27367821

[56] Peel NM, Paul SK, Cameron ID, Crotty M, Kurrle SE, Gray LC. Promoting activity in geriatric rehabilitation: a randomized controlled trial of Accelerometry. PLoS One. 2016;11(8). 10.1371/journal.pone.0160906. 10.1371/journal.pone.0160906 PMC5001632 27564857

[57] Atkins A, Cannell J, Barr C. Pedometers alone do not increase mobility in inpatient rehabilitation: a randomized controlled trial. Clin Rehabil. 2019;33(8). 10.1177/0269215519838312. 10.1177/0269215519838312 30955362

[58] Houle J, Doyon O, Vadeboncoeur N, Turbide G, Diaz A, Poirier P. Effectiveness of a pedometer-based program using a socio-cognitive intervention on physical activity and quality of life in a setting of cardiac rehabilitation. Can J Cardiol. 2012;28(1). 10.1016/j.cjca.2011.09.020. 10.1016/j.cjca.2011.09.020 22177854

[59] Atkins D, Best D, Briss PA, Eccles M, Falck-Ytter Y, Flottorp S, et al. Grading quality of evidence and strength of recommendations. BMJ (Clinical research ed). 2004;328(7454). 10.1136/bmj.328.7454.1490. 10.1136/bmj.328.7454.1490 PMC428525 15205295

[60] Eisele A, Schagg D, Krämer LV, Bengel J, Göhner W. Behaviour change techniques applied in interventions to enhance physical activity adherence in patients with chronic musculoskeletal conditions: a systematic review and meta-analysis. Patient Educ Couns. 2019;102(1). 10.1016/j.pec.2018.09.018. 10.1016/j.pec.2018.09.018 30279029

[61] Bishop FL, Fenge-Davies AL, Kirby S, Geraghty AW. Context effects and behaviour change techniques in randomised trials: a systematic review using the example of trials to increase adherence to physical activity in musculoskeletal pain. Psychol Health. 2015;30(1). 10.1080/08870446.2014.953529. 10.1080/08870446.2014.953529 25109300

[62] Chia GLC, Anderson A, McLean LA. Behavior change techniques incorporated in fitness trackers: content analysis. JMIR Mhealth Uhealth. 2019;7(7). 10.2196/12768. 10.2196/12768 PMC6683653 31339101

[63] Low CA. Harnessing consumer smartphone and wearable sensors for clinical cancer research. npj Digital Medicine. 2020;3(1). 10.1038/s41746-020-00351-x. 10.1038/s41746-020-00351-x PMC7591557 33134557

[64] Direito A, Carraça E, Rawstorn J, Whittaker R, Maddison R. mHealth technologies to influence physical activity and sedentary behaviors: behavior change techniques, systematic review and Meta-analysis of randomized controlled trials. Ann Behav Med. 2017;51(2). 10.1007/s12160-016-9846-0. 10.1007/s12160-016-9846-0 27757789

[65] Samdal GB, Eide GE, Barth T, Williams G, Meland E. Effective behaviour change techniques for physical activity and healthy eating in overweight and obese adults; systematic review and meta-regression analyses. Int J Behav Nutr Phys Act. 2017;14(1). 10.1186/s12966-017-0494-y. 10.1186/s12966-017-0494-y PMC5370453 28351367

