# Supplementary material for: The effectiveness of physical activity interventions using activity trackers during or after inpatient care: a systematic review and meta-analysis of randomized controlled trials
Source: Int J Behav Nutr Phys Act. 2022 May 23;19:59. doi: 10.1186/s12966-022-01261-9 (PMC9125831; doi:10.1186/s12966-022-01261-9)

**Electronic Supplementary Material Table S1.** Systematic literature review search terms and strategy

| **Search strategy for PubMed (3 March 2021)** | | |
| --- | --- | --- |
| **Search** | **Query** | **Results** |
| **#4** | **#3 NOT (("Adolescent"[Mesh] OR "Child"[Mesh] OR "Infant"[Mesh] OR "adolescen*"[tiab] OR "child*"[tiab] OR "schoolchild*"[tiab] OR "infant*"[tiab] OR "girl*"[tiab] OR "boy"[tiab] OR "boys"[tiab] OR "teen"[tiab] OR "teens"[tiab] OR "teenager*"[tiab] OR "youth*"[tiab] OR "pediatr*"[tiab] OR "paediatr*"[tiab] OR "puber*"[tiab]) NOT ("Adult"[Mesh] OR "adult*"[tiab] OR "man"[tiab] OR "men"[tiab] OR "woman"[tiab] OR "women"[tiab]))** | [**5,433**](https://pubmed.ncbi.nlm.nih.gov/?term=%233+NOT+%28%28%22Adolescent%22%5BMesh%5D+OR+%22Child%22%5BMesh%5D+OR+%22Infant%22%5BMesh%5D+OR+%22adolescen%2A%22%5Btiab%5D+OR+%22child%2A%22%5Btiab%5D+OR+%22schoolchild%2A%22%5Btiab%5D+OR+%22infant%2A%22%5Btiab%5D+OR+%22girl%2A%22%5Btiab%5D+OR+%22boy%22%5Btiab%5D+OR+%22boys%22%5Btiab%5D+OR+%22teen%22%5Btiab%5D+OR+%22teens%22%5Btiab%5D+OR+%22teenager%2A%22%5Btiab%5D+OR+%22youth%2A%22%5Btiab%5D+OR+%22pediatr%2A%22%5Btiab%5D+OR+%22paediatr%2A%22%5Btiab%5D+OR+%22puber%2A%22%5Btiab%5D%29+NOT+%28%22Adult%22%5BMesh%5D+OR+%22adult%2A%22%5Btiab%5D+OR+%22man%22%5Btiab%5D+OR+%22men%22%5Btiab%5D+OR+%22woman%22%5Btiab%5D+OR+%22women%22%5Btiab%5D%29%29&sort=date&size=200&ac=no) |
| **#3** | **#1 AND #2** | **5,778** |
| **#2** | **"Accelerometry"[Mesh] OR "Wearable Electronic Devices"[Mesh:NoExp] OR "Fitness Trackers"[Mesh] OR "fitness tracker*"[tiab] OR "activity tracker*"[tiab] OR "wearable*"[tiab] OR "activity track*"[tiab] OR "physical activity monitor*"[tiab] OR "acceleromet*"[tiab] OR "actigraph*"[tiab] OR "activpal"[tiab] OR "actical"[tiab] OR "fitbit*"[tiab] OR "smartwatch*"[tiab] OR "smart watch*"[tiab] OR "pedomet*"[tiab] OR "movemonitor*"[tiab] OR ""move monitor""[tiab] OR "activ8"[tiab] OR "stepcount*"[tiab] OR "step count*"[tiab] OR "sensor monitor*"[tiab]** | **44,115** |
| **#1** | **"Postoperative Period"[Mesh:NoExp] OR "Postoperative Care"[Mesh] OR "Postoperative Complications"[Mesh:NoExp] OR "Pain, Postoperative"[Mesh:NoExp] OR "Hospitalization"[Mesh] OR "Rehabilitation Centers"[Mesh:NoExp] OR "Hospital Departments"[Mesh] OR "Academic Medical Centers"[Mesh] OR "Hospital Units"[Mesh] OR "Hospitals"[Mesh:NoExp] OR "Inpatients"[Mesh] OR "Recovery of Function"[Mesh] OR "Convalescence"[Mesh] OR "recover*"[tiab] OR "convalescenc*"[tiab] OR "inpatient*"[tiab] OR "medical center*"[tiab] OR "postoperative*"[tiab] OR "post operat*"[tiab] OR "stay length*"[tiab] OR "length of stay*"[tiab] OR "hospital stay*"[tiab] OR "patient admission*"[tiab] OR "patient discharge*"[tiab] OR "discharge planning*"[tiab] OR "patient handoff*"[tiab] OR "patient hand over*"[tiab] OR "patient sign out*"[tiab] OR "patient signout*"[tiab] OR "patient signover*"[tiab] OR "patient hand off*"[tiab] OR "patient sign out*"[tiab] OR "patient handover*"[tiab] OR "readmission*"[tiab] OR "patient transfer*"[tiab] OR "patient transition*"[tiab] OR "care transition*"[tiab] OR "transition of care*"[tiab] OR "rehabilitati*"[tiab] OR "hospital"[tiab] OR "hospitals"[tiab] OR "hospitalisation*"[tiab] OR "hospitalization*"[tiab] OR "after surg*"[tiab]** | **3,293,661** |
| **Search strategy for Embase.com (3 March 2021)** | | |
| **No.** | **Query** | **Results** |
| **#5** | #4 NOT ('conference abstract'/it OR 'conference paper'/it OR 'conference review'/it) | **4640** |
| **#4** | #3 NOT (('adolescent'/exp OR 'child'/exp OR adolescent*:ti,ab OR child*:ti,ab OR schoolchild*:ti,ab OR infant*:ti,ab OR girl*:ti,ab OR boy*:ti,ab OR teen:ti,ab OR teens:ti,ab OR teenager*:ti,ab OR youth*:ti,ab OR pediatr*:ti,ab OR paediatr*:ti,ab OR puber*:ti,ab) NOT ('adult'/exp OR 'aged'/exp OR 'middle aged'/exp OR adult*:ti,ab OR man:ti,ab OR men:ti,ab OR woman:ti,ab OR women:ti,ab)) | **7510** |
| **#3** | #1 AND #2 | **8039** |
| **#2** | 'accelerometry'/exp OR 'wearable computer'/exp OR 'activity tracker'/exp OR 'fitness tracker*':ti,ab,kw OR wearable*:ti,ab,kw OR 'activity track*':ti,ab,kw OR 'physical activity monitor*':ti,ab,kw OR acceleromet*:ti,ab,kw OR actigraph*:ti,ab,kw OR activpal:ti,ab,kw OR actical:ti,ab,kw OR fitbit*:ti,ab,kw OR smartwatch*:ti,ab,kw OR 'smart watch*':ti,ab,kw OR pedomet*:ti,ab,kw OR movemonitor*:ti,ab,kw OR 'move monitor':ti,ab,kw OR activ8:ti,ab,kw OR stepcount*:ti,ab,kw OR 'step count*':ti,ab,kw OR 'sensor monitor*':ti,ab,kw | **56249** |
| **#1** | 'postoperative period'/de OR 'postoperative care'/exp OR 'postoperative complication'/de OR 'postoperative pain'/exp OR 'hospitalization'/exp OR 'rehabilitation center'/exp OR 'hospital department'/exp OR 'university hospital'/exp OR 'hospital subdivisions and components'/de OR 'ward'/exp OR 'operating room'/exp OR 'hospital'/de OR 'hospital patient'/de OR 'aged hospital patient'/exp OR 'convalescence'/exp OR ((recover*:ti,ab,kw OR convalescenc*:ti,ab,kw OR inpatient*:ti,ab,kw OR medical:ti,ab,kw) AND center*:ti,ab,kw) OR postoperative*:ti,ab,kw OR 'post operat*':ti,ab,kw OR 'stay length*':ti,ab,kw OR 'length of stay*':ti,ab,kw OR 'hospital stay*':ti,ab,kw OR 'patient admission*':ti,ab,kw OR 'patient discharge*':ti,ab,kw OR 'discharge planning*':ti,ab,kw OR 'patient handoff*':ti,ab,kw OR 'patient hand over*':ti,ab,kw OR 'patient signout*':ti,ab,kw OR 'patient signover*':ti,ab,kw OR 'patient hand off*':ti,ab,kw OR 'patient sign out*':ti,ab,kw OR 'patient handover*':ti,ab,kw OR readmission*:ti,ab,kw OR 'patient transfer*':ti,ab,kw OR 'patient transition*':ti,ab,kw OR 'care transition*':ti,ab,kw OR 'transition of care*':ti,ab,kw OR rehabilitati*:ti,ab,kw OR hospital:ti,ab,kw OR hospitals:ti,ab,kw OR hospitalisation*:ti,ab,kw OR hospitalization*:ti,ab,kw OR 'after surg*':ti,ab,kw | **4071513** |
| **Search strategy for Ebsco/CINAHL (3 March 2021)** | | |
| **#** | **Query** | **Results** |
| **S4** | S3 NOT ((MH ("Adolescence" OR "Child+") OR TI (adolescen* OR child* OR schoolchild* OR infant* OR girl* OR boy* OR teen OR teens OR teenager* OR youth* OR pediatr* OR paediatr* OR puber*) OR AB (adolescen* OR child* OR schoolchild* OR infant* OR girl* OR boy* OR teen OR teens OR teenager* OR youth* OR pediatr* OR paediatr* OR puber*)) NOT (MH ("Adult+") OR TI (adult* OR man OR men OR woman OR women) OR AB (adult* OR man OR men OR woman OR women))) | **2,357** |
| **S3** | S1 AND S2 | **2,545** |
| **S2** | (MH "Accelerometry+") OR (MH "Accelerometers") OR (MH "Pedometers") OR (MH "Fitness Trackers") OR TI (“fitness tracker*” OR wearable* OR “activity track*” OR “physical activity monitor*” OR acceleromet* OR actigraph* OR activpal OR actical OR fitbit* OR smartwatch* OR “smart watch*” OR pedomet* OR movemonitor* OR “move monitor” OR activ8 OR stepcount* OR “step count*” OR “sensor monitor*”) OR AB (“fitness tracker*” OR wearable* OR “activity track*” OR “physical activity monitor*” OR acceleromet* OR actigraph* OR activpal OR actical OR fitbit* OR smartwatch* OR “smart watch*” OR pedomet* OR movemonitor* OR “move monitor” OR activ8 OR stepcount* OR “step count*” OR “sensor monitor*”) OR KW (“fitness tracker*” OR wearable* OR “activity track*” OR “physical activity monitor*” OR acceleromet* OR actigraph* OR activpal OR actical OR fitbit* OR smartwatch* OR “smart watch*” OR pedomet* OR movemonitor* OR “move monitor” OR activ8 OR stepcount* OR “step count*” OR “sensor monitor*”) | **17,890** |
| **S1** | (MH "Postoperative Care") OR (MH "Postoperative Pain") OR (MH "Postoperative Complications") OR (MH "Hospitalization") OR (MH "Rehabilitation Centers") OR (MH "Health Facility Departments+") OR (MH "Hospitals, Public+") OR (MH "Hospitals+") OR (MH "Hospitals, Special+") OR (MH "Hospital Units+") OR (MH "Aged, Hospitalized") OR (MH "Patients") OR (MH "Inpatients") OR (MH "Stroke Patients") OR (MH "Rehabilitation Patients") OR (MH "Surgical Patients") OR (MH "Cancer Patients") OR (MH "Cardiac Patients") OR (MH "Emergency Patients") OR (MH "Recovery") OR (MH "Hand Off (Patient Safety)") OR (MH "Transfer, Discharge") OR (MH "Transfer, Discharge") OR (MH "Discharge Planning+") OR (MH "Patient Discharge") OR TI (recover* OR convalescenc* OR inpatient* OR medical center* OR postoperative* OR “post operat*” OR “stay length*” OR “length of stay*” OR “hospital stay*” OR “patient admission*” OR “patient discharge*” OR “discharge planning*” OR “patient handoff*” OR “patient hand over*” OR “patient sign out*” OR “patient signout*” OR “patient signover*” OR “patient hand off*” OR “patient sign out*” OR “patient handover*” OR readmission* OR “patient transfer*” OR “patient transition*” OR “care transition*” OR “transition of care*” OR rehabilitati* OR hospital OR hospitals OR hospitalisation* OR hospitalization* OR “after surg*”) OR AB (recover* OR convalescenc* OR inpatient* OR medical center* OR postoperative* OR “post operat*” OR “stay length*” OR “length of stay*” OR “hospital stay*” OR “patient admission*” OR “patient discharge*” OR “discharge planning*” OR “patient handoff*” OR “patient hand over*” OR “patient sign out*” OR “patient signout*” OR “patient signover*” OR “patient hand off*” OR “patient sign out*” OR “patient handover*” OR readmission* OR “patient transfer*” OR “patient transition*” OR “care transition*” OR “transition of care*” OR rehabilitati* OR hospital OR hospitals OR hospitalisation* OR hospitalization* OR “after surg*”) OR KW (recover* OR convalescenc* OR inpatient* OR medical center* OR postoperative* OR “post operat*” OR “stay length*” OR “length of stay*” OR “hospital stay*” OR “patient admission*” OR “patient discharge*” OR “discharge planning*” OR “patient handoff*” OR “patient hand over*” OR “patient sign out*” OR “patient signout*” OR “patient signover*” OR “patient hand off*” OR “patient sign out*” OR “patient handover*” OR readmission* OR “patient transfer*” OR “patient transition*” OR “care transition*” OR “transition of care*” OR rehabilitati* OR hospital OR hospitals OR hospitalisation* OR hospitalization* OR “after surg*”) | **1,104,113** |
| **Search strategy for Ebsco/SportDiscus (3 March 2021)** | | |
| **#** | **Query** | **Results** |
| **S4** | S1 AND S2 AND S3 | **222** |
| **S3** | DE "YOUNG adults" OR DE "OLDER people" OR TI (adult* OR man OR men OR woman OR women) OR AB (adult* OR man OR men OR woman OR women) OR KW (adult* OR man OR men OR woman OR women) | **254,522** |
| **S2** | DE "ACCELEROMETERS" OR DE "SPEEDOMETERS" OR DE "PEDOMETERS" OR TI (“fitness tracker*” OR wearable* OR “activity track*” OR “physical activity monitor*” OR acceleromet* OR actigraph* OR activpal OR actical OR fitbit* OR smartwatch* OR “smart watch*” OR pedomet* OR movemonitor* OR “move monitor” OR activ8 OR stepcount* OR “step count*” OR “sensor monitor*”) OR AB (“fitness tracker*” OR wearable* OR “activity track*” OR “physical activity monitor*” OR acceleromet* OR actigraph* OR activpal OR actical OR fitbit* OR smartwatch* OR “smart watch*” OR pedomet* OR movemonitor* OR “move monitor” OR activ8 OR stepcount* OR “step count*” OR “sensor monitor*”) OR KW (“fitness tracker*” OR wearable* OR “activity track*” OR “physical activity monitor*” OR acceleromet* OR actigraph* OR activpal OR actical OR fitbit* OR smartwatch* OR “smart watch*” OR pedomet* OR movemonitor* OR “move monitor” OR activ8 OR stepcount* OR “step count*” OR “sensor monitor*”) | **9,517** |
| **S1** | DE "CONVALESCENCE" OR DE "HOSPITALS" OR DE "HOSPITAL care" OR DE "MEDICAL rehabilitation" OR DE "CLINICS" OR DE "MEDICAL centers" OR DE "HEALTH facilities" OR DE "PATIENTS" OR DE "CANCER patients" OR DE "TRANSFER of medical care" OR TI (recover* OR convalescenc* OR inpatient* OR medical center* OR postoperative* OR “post operat*” OR “stay length*” OR “length of stay*” OR “hospital stay*” OR “patient admission*” OR “patient discharge*” OR “discharge planning*” OR “patient handoff*” OR “patient hand over*” OR “patient sign out*” OR “patient signout*” OR “patient signover*” OR “patient hand off*” OR “patient sign out*” OR “patient handover*” OR readmission* OR “patient transfer*” OR “patient transition*” OR “care transition*” OR “transition of care*” OR rehabilitati* OR hospital OR hospitals OR hospitalisation* OR hospitalization* OR “after surg*”) OR AB (recover* OR convalescenc* OR inpatient* OR medical center* OR postoperative* OR “post operat*” OR “stay length*” OR “length of stay*” OR “hospital stay*” OR “patient admission*” OR “patient discharge*” OR “discharge planning*” OR “patient handoff*” OR “patient hand over*” OR “patient sign out*” OR “patient signout*” OR “patient signover*” OR “patient hand off*” OR “patient sign out*” OR “patient handover*” OR readmission* OR “patient transfer*” OR “patient transition*” OR “care transition*” OR “transition of care*” OR rehabilitati* OR hospital OR hospitals OR hospitalisation* OR hospitalization* OR “after surg*”) OR KW (recover* OR convalescenc* OR inpatient* OR medical center* OR postoperative* OR “post operat*” OR “stay length*” OR “length of stay*” OR “hospital stay*” OR “patient admission*” OR “patient discharge*” OR “discharge planning*” OR “patient handoff*” OR “patient hand over*” OR “patient sign out*” OR “patient signout*” OR “patient signover*” OR “patient hand off*” OR “patient sign out*” OR “patient handover*” OR readmission* OR “patient transfer*” OR “patient transition*” OR “care transition*” OR “transition of care*” OR rehabilitati* OR hospital OR hospitals OR hospitalisation* OR hospitalization* OR “after surg*”) | **122,050** |
| **Search strategy for Clarivate Analytics/Web of Science Core Collection (3 March 2021)** | | |
| **Set** | **Query** | **Results** |
| **#4** | #3 AND #2 AND #1 | **1,568** |
| **#3** | **TOPIC:** (“adult*” OR “man” OR “men” OR “woman” OR “women”) | **3,530,102** |
| **#2** | **TOPIC:** (“fitness tracker*” OR “wearable*” OR “activity track*” OR “physical activity monitor*” OR “acceleromet*” OR “actigraph*” OR “activpal” OR “actical” OR “fitbit*” OR “smartwatch*” OR “smart watch*” OR “pedomet*” OR “movemonitor*” OR “move monitor” OR “activ8” OR “stepcount*” OR “step count*” OR “sensor monitor*”) | **69,518** |
| **#1** | **TOPIC:** (“recover*” OR “convalescenc*” OR “inpatient*” OR “medical center*” OR “postoperative*” OR “post operat*” OR “stay length*” OR “length of stay*” OR “hospital stay*” OR “patient admission*” OR “patient discharge*” OR “discharge planning*” OR “patient handoff*” OR “patient hand over*” OR “patient sign out*” OR “patient signout*” OR “patient signover*” OR “patient hand off*” OR “patient sign out*” OR “patient handover*” OR “readmission*” OR “patient transfer*” OR “patient transition*” OR “care transition*” OR “transition of care*” OR “rehabilitati*” OR “hospital” OR “hospitals” OR “hospitalisation*” OR “hospitalization*” OR “after surg*”) | **2,827,859** |

**Electronic Supplementary Material Table S2.** Data extraction Behavioural Change Techniques (BCTs)

| Auteur (year) | **Total** | **1.1 Goal setting (behavior)** | **1.2 Problem solving** | **1.3 Goal Setting (outcome)** | **1.4 Action planning** | **1.5 Review behavior goal(s)** | **1.6 Discrepancy betw current behavior and goal** | **2.2 Feedback on behaviour** | **2.3 Self-monitoring of behavior** | **2.4 Self-monitoring of outcome(s) of behaviour** | **2.7 Feedback on outcome(s) of behavior** |
| --- | --- | --- | --- | --- | --- | --- | --- | --- | --- | --- | --- |
| Atkins (2019) | 2 | 0 | 0 | 0 | 0 | 0 | 0 | 1 | 1 | 0 | 0 |
| Brandes (2018) | 6 | 1 | 0 | 0 | 1 | 0 | 0 | 1 | 0 | 0 | 0 |
| Christiansen (2020) | 5 | 1 | 0 | 0 | 0 | 1 | 0 | 1 | 1 | 0 | 0 |
| Creel_Ped (2016) | 4 | 1 | 0 | 0 | 0 | 0 | 0 | 1 | 1 | 0 | 0 |
| Creel_Couns (2016) | 10 | 0 | 1 | 1 | 1 | 0 | 0 | 1 | 0 | 1 | 1 |
| Dorsch (2016) | 2 | 0 | 0 | 0 | 0 | 1 | 0 | 1 | 0 | 0 | 0 |
| Frederix (2015) | 6 | 1 | 0 | 0 | 1 | 0 | 0 | 1 | 1 | 0 | 0 |
| Hassett (2020) | 9 | 1 | 1 | 0 | 1 | 0 | 0 | 1 | 0 | 0 | 0 |
| Hornikx (2015) | 7 | 1 | 1 | 0 | 0 | 0 | 0 | 1 | 1 | 0 | 0 |
| Houle (2011) | 12 | 1 | 1 | 0 | 1 | 0 | 0 | 1 | 1 | 0 | 0 |
| Izawa (2005) | 10 | 1 | 1 | 0 | 0 | 0 | 0 | 1 | 1 | 1 | 1 |
| Izawa (2012) | 9 | 1 | 1 | 1 | 0 | 0 | 0 | 1 | 1 | 0 | 0 |
| Kanai (2018) | 9 | 1 | 0 | 0 | 1 | 1 | 0 | 1 | 1 | 0 | 0 |
| Lawrie (2018) | 5 | 1 | 0 | 0 | 1 | 0 | 1 | 1 | 0 | 0 | 0 |
| Mansfield (2015) | 1 | 0 | 0 | 0 | 0 | 0 | 0 | 1 | 0 | 0 | 0 |
| Mehta_int A (2020) | 4 | 0 | 0 | 0 | 0 | 0 | 0 | 1 | 1 | 0 | 0 |
| Mehta_Int B (2020) | 9 | 1 | 0 | 0 | 1 | 0 | 0 | 1 | 1 | 0 | 0 |
| Moller (2015) | 6 | 1 | 1 | 0 | 0 | 0 | 0 | 1 | 1 | 0 | 0 |
| Peel (2016) | 5 | 0 | 0 | 0 | 1 | 1 | 1 | 1 | 0 | 0 | 0 |
| Pol (2019) | 2 | 0 | 0 | 0 | 0 | 0 | 0 | 1 | 0 | 0 | 0 |
| Van der Meij (2018) | 8 | 0 | 0 | 1 | 1 | 0 | 1 | 1 | 1 | 1 | 0 |
| Van der Walt (2018) | 5 | 1 | 0 | 0 | 1 | 0 | 0 | 1 | 1 | 0 | 0 |
| Wolk (2019) | 5 | 1 | 0 | 0 | 1 | 0 | 0 | 1 | 1 | 0 | 0 |
| **Total** |  | **15/23** | **7/23** | **3/23** | **12/23** | **4/23** | **3/23** | **23/23** | **15/23** | **3/23** | **2/23** |

| **Table continued** | | | | | | | | | | |
| --- | --- | --- | --- | --- | --- | --- | --- | --- | --- | --- |
| Auteur (year) | **3.1 Social Support (unspecified)** | **4.1 Instructions on how to perform the behavior** | **5.1 Information about health consequences** | **5.3 Information about social and environmental consequences** | **6.2 Social comparison** | **7.1 Prompts/cues** | **8.7 graded tasks** | **10.3 Nonspecific reward** | **10.4 Social reward** | **12.5 Adding objects to the environment** |
| Atkins (2019) | 0 | 0 | 0 | 0 | 0 | 0 | 0 | 0 | 0 | 0 |
| Brandes (2018) | 1 | 0 | 0 | 0 | 0 | 0 | 1 | 0 | 0 | 1 |
| Christiansen (2020) | 0 | 0 | 0 | 0 | 0 | 0 | 0 | 0 | 0 | 1 |
| Creel_Ped (2016) | 0 | 0 | 0 | 0 | 0 | 0 | 0 | 0 | 0 | 1 |
| Creel_Couns (2016) | 1 | 1 | 0 | 1 | 0 | 0 | 0 | 0 | 0 | 1 |
| Dorsch (2016) | 0 | 0 | 0 | 0 | 0 | 0 | 0 | 0 | 0 | 0 |
| Frederix (2015) | 0 | 0 | 0 | 0 | 0 | 1 | 1 | 0 | 0 | 0 |
| Hassett (2020) | 1 | 1 | 0 | 0 | 0 | 1 | 1 | 0 | 0 | 1 |
| Hornikx (2015) | 1 | 0 | 0 | 0 | 0 | 1 | 0 | 0 | 0 | 1 |
| Houle (2011) | 1 | 1 | 1 | 0 | 1 | 1 | 1 | 0 | 0 | 1 |
| Izawa (2005) | 1 | 0 | 0 | 0 | 0 | 0 | 1 | 0 | 1 | 1 |
| Izawa (2012) | 1 | 0 | 0 | 0 | 0 | 0 | 1 | 0 | 1 | 1 |
| Kanai (2018) | 1 | 0 | 0 | 0 | 0 | 0 | 1 | 0 | 1 | 1 |
| Lawrie (2018) | 0 | 0 | 0 | 0 | 0 | 0 | 1 | 0 | 0 | 0 |
| Mansfield (2015) | 0 | 0 | 0 | 0 | 0 | 0 | 0 | 0 | 0 | 0 |
| Mehta_int A (2020) | 0 | 0 | 0 | 0 | 0 | 1 | 0 | 0 | 0 | 1 |
| Mehta_Int B (2020) | 1 | 0 | 0 | 0 | 0 | 1 | 1 | 1 | 0 | 1 |
| Moller (2015) | 1 | 0 | 0 | 0 | 0 | 0 | 0 | 0 | 0 | 1 |
| Peel (2016) | 0 | 0 | 0 | 0 | 0 | 0 | 1 | 0 | 0 | 0 |
| Pol (2019) | 0 | 0 | 0 | 0 | 0 | 0 | 0 | 0 | 0 | 1 |
| Van der Meij (2018) | 1 | 0 | 0 | 0 | 0 | 0 | 0 | 0 | 0 | 1 |
| Van der Walt (2018) | 0 | 0 | 0 | 0 | 0 | 0 | 1 |  | 0 | 0 |
| Wolk (2019) | 0 | 0 | 0 | 0 | 0 | 0 | 1 |  | 0 | 0 |
| **Total** | **12/23** | **3/23** | **1/23** | **1/23** | **1/23** | **6/23** | **12/23** | **1/23** | **3/23** | **15/23** |

**Electronic Supplementary Material Table S3.** Certainty of evidence for primary outcomes

|  | Quality assessment | | | | | | No. of participants | | Summary of Findings | |
| --- | --- | --- | --- | --- | --- | --- | --- | --- | --- | --- |
| No. of Studies | Design | Methodological limitations of the studies | Inconsistency | Indirectness | Imprecision | Other modifying factors | Intervention | Control | Relative Effect  (95% CI) | Certainty of evidence  Importance* |
| Physical activity | | | | | | | | | | |
| 13 | RCT | No serious limitations^a^ | Some inconsistency^b^ | No serious indirectness^c^ | No serious imprecision^d^ | None | 729 | 706 | SMD 0.34 (0.12 – 0.56) | Moderate  ⊕⊕⊕O |
| Physical functioning (performance based) | | | | | | | | | | |
| 13 | RCT | No serious limitations^a^ | No serious inconsistency | Serious indirectness^e^ | No serious imprecision | None | 696 | 719 | SMD 0.09  (-0.02 – 0.20) | Low  ⊕⊕OO |
| Physical functioning (patient reported outcome) | | | | | | | | | | |
| 3 | RCT | No serious limitations | Some inconsistency^f^ | Serious indirectness^e^ | Serious imprecision^h^ | None | 262 | 280 | SMD 0.15  (-0.18 – 0.47) | Very Low  ⊕OOO |
| *RCT* Randomized controlled trial, *SMD* Standardized Mean Difference  *Commonly used symbols to describe certainty in evidence in evidence profiles: high certainty ⊕⊕⊕⊕, moderate certainty ⊕⊕⊕O, low certainty ⊕⊕OO and very low certainty ⊕OOO.  ^a^Not all studies reported whether they perform an “intention-to-treat-analysis”, this might have caused under- or overestimation of the effect sizes.  ^b^There was some variation in direction and magnitude across the different trials. Overall the results showed a small positive effect on PA. The direction of the effect size of the study of Wolk (subgroup open surgery, 2019) was inconsistent compared to the other trials. This inconsistency might be caused by the use of a too low set step target in this subgroup of the study. The study of Izawa (2012) showed an larger effect size compared to the other trials. This resulted in a higher level of statistical heterogeneity (I^2^=73%)  ^c.^The patients, interventions and control groups in all studies provide direct evidence to the clinical research question. Despite the variability in the intervention characteristics, we judged the evidence to have no serious indirectness.  ^d^Some studies showed higher confidence intervals due to a smaller sample size. However, due to the large number of included participants judged the evidence to have no serious imprecision.  ^e^There was major uncertainty about the directness in outcome, because there was a high variability in outcomes measures of performance based physical functioning  ^f^The direction of the effect of one trial (Van der Meij, 2018) varied from the other 2 trials.  ^g^Due to the high variability in outcome measures, there was major uncertainty about the directness.  ^h^The total number of patients included in all trials was low and the overall effect sizes showed a wide confidence interval. | | | | | | | | | | |

**Electronic Supplementary Material Figure S1.** Funnel plot for the outcome physical activity

**
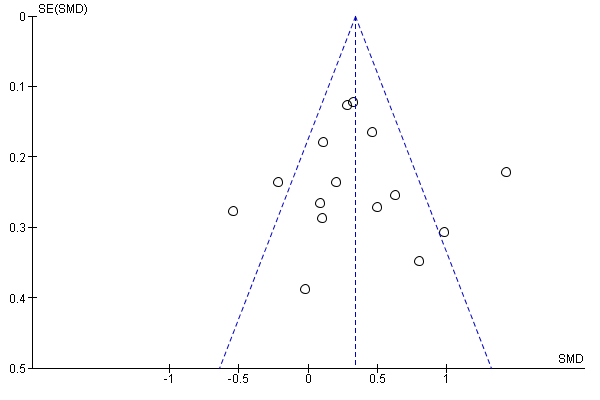
**

**Electronic Supplementary Material Figure S2.** Funnel plot for the outcome performance based physical functioning

**
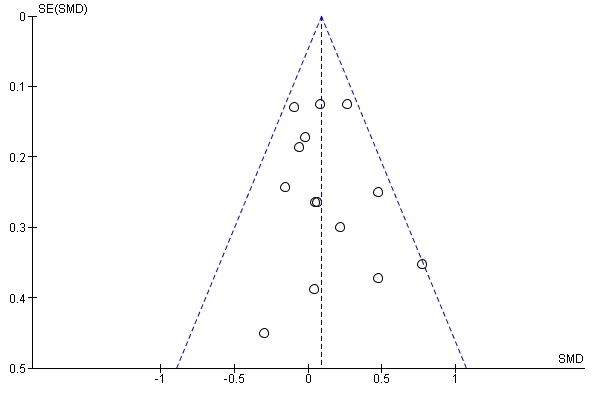
**

**Electronic Supplementary Material Figure S3.** Forest plot for the subgroup analyses between interventions with ≥ 7 BCT’s vs. interventions with less than 7 BCT’s on the outcome physical activity

**
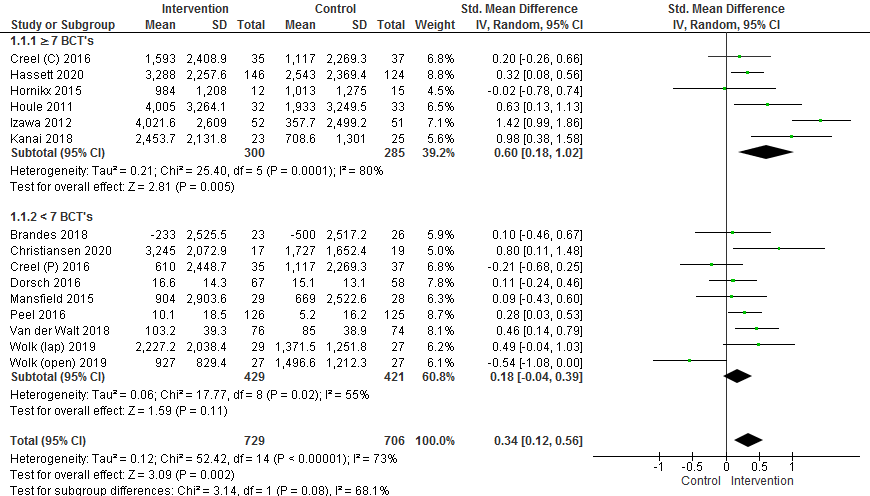
**

**Electronic Supplementary Material Figure S4.** Forest plot for the subgroup analyses between interventions with a theoretical model used vs. interventions without a theoretical model used on the outcome physical activity

**
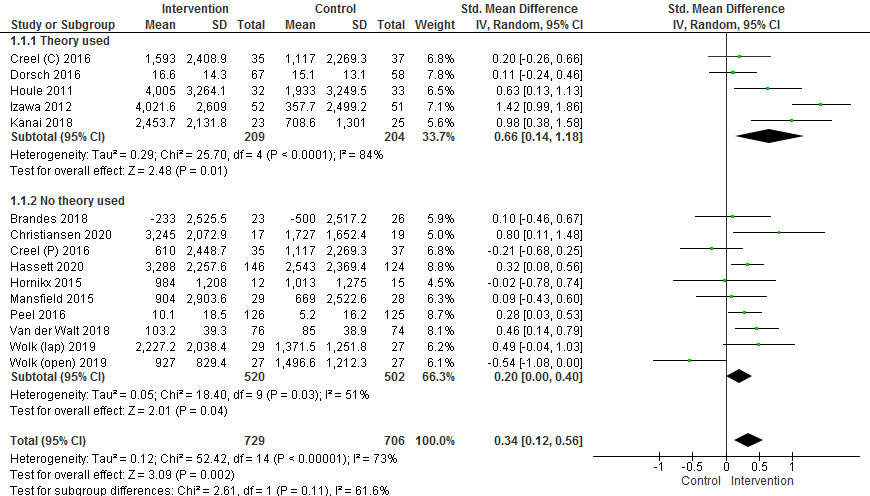
**

**Electronic Supplementary Material Figure S5.** Forest plot for the subgroup analyses between interventions with coaching by a health professional (HP) vs. interventions without coaching by a HP on the outcome physical activity


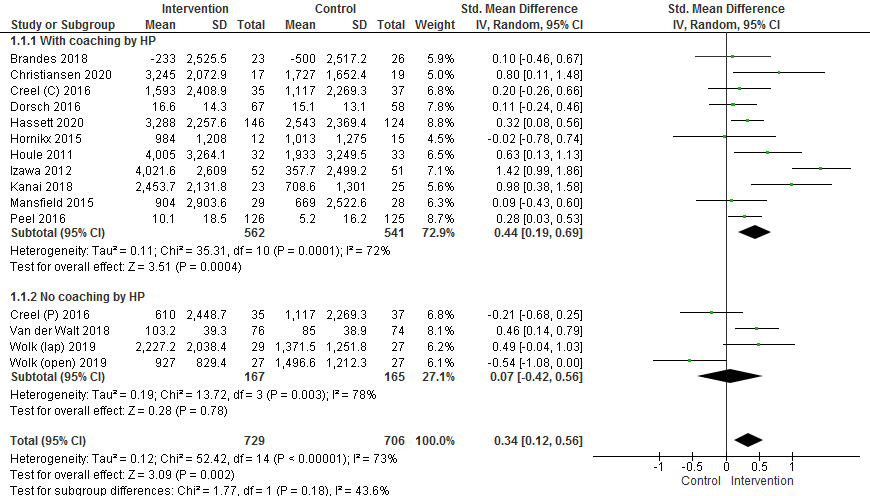

Supplement: Supplementary file 1 — Additional file 1. [file 12966_2022_1261_MOESM1_ESM.docx]
